# Effects of melatonin treatment on germination, growth and physiological characteristics under drought stress in foxtail millet

**DOI:** 10.3389/fpls.2025.1601253

**Published:** 2025-06-06

**Authors:** Chaomin Meng, Cheng Wang, Haojie Guo, Jinyue Wang, Jingchen Yu

**Affiliations:** College of Agriculture, Henan University of Science and Technology, Luoyang, China

**Keywords:** melatonin, foxtail millet, germination, drought stress, photosynthetic characteristics, physiological and biochemical characteristics

## Abstract

Melatonin is a key antioxidant that not only promotes seed germination, but also slows cellular aging and enhances stress resistance. However, the specific mechanisms underlying melatonin’s effects in foxtail millet (*Setaria italica* L.) are not yet fully understood. In this study, three varieties of millet - ‘Yugu 18’, ‘Jigu 38’ and ‘Changnong 35’ - served as test materials. We examined the influence of varying melatonin concentrations (0, 5, 25, 50, 75, 100, 125, 150, 200μmol·L^-1^) on germination, growth, photosynthetic properties, antioxidant capabilities, and osmoregulatory substance content of millet under drought conditions, using a pot experiment with PEG 6000. The results revealed that different melatonin concentrations exhibited a “low promotion and high elevation” pattern for seed germination, seedling length, and root length. At equivalent concentrations, the inhibitory effect on seed germination was strongest for ‘Yugu 18’, while it was weakest for ‘Jigu 38’ and ‘Changnong 35’. Notably, compared to no melatonin treatment, the 75μmol·L^-1^ melatonin treatment most significantly increased photosynthesis rate, intercellular CO_2_ concentration, chlorophyll content, CAT activity, and POD activity by 27.21%, 13.15%, 74.79%, 18.75% and 39.58%, respectively, while significantly reducing MDA content by 32.61%. Based on the weight calculation of principal components, the activities of POD and SOD, along with the contents of Ci, GSH, and soluble protein, were identified as the major contributors to the first five indices in the order: POD activity > Ci > GSH content > soluble protein content > SOD activity. The drought resistance of the nine treatments under drought stress was ranked: MT100 > MT75 > MT150 > MT125 > MT50 > CK > MT200 > MT25 > MT5. For the Jigu 38 cultivar, the optimal melatonin concentration was established at 75 μmol·L^-1^, while for Yugu 18 and Changnong 35, it was 100 μmol·L^-1^. This concentration effectively modulated the photosynthetic capacity, antioxidant enzyme activity, and osmotic ability of millet seedlings, thereby augmenting their drought resistance. This study offers a scientific basis for comprehending the mechanism by which melatonin mitigates drought stress in diverse millet varieties.

## 1 Introduction

Water is a critical ecological component for the growth and development of crops. However, recent global warming has led to the expansion of seasonal dry areas, severely impacting agricultural production. Of the world’s 1.4 billion hm^2^ of cultivated land, 43% is subjected to drought and semi-drought conditions, causing approximately 50% loss in crop yield – a figure surpassing that of all other natural disasters combined (Lv et al., 2021). Drought stress, a multidimensional burden, triggers the buildup of peroxides and reactive oxygen species (ROS) in plants, disrupting the balance between ROS production and eradication. This imbalance induces cell membrane peroxidation, protein oxidation, and alterations in membrane permeability. It also increases malondialdehyde (MDA) content while reducing osmotic regulation and antioxidant capacity, eventually leading to plant death and stunting their normal development (Li et al., 2019; Ye et al., 2016). Research indicates that under drought stress, plants actively accumulate specific osmoregulatory substances to maintain cellular osmotic pressure and ensure the normal functioning of water physiological metabolism (Mohammad et al., 2021). Concurrently, plants activate their intrinsic antioxidant enzyme system to counteract excessive reactive oxygen species and shield cell membranes from harm (Lai and Luo, 2019; Huang et al., 2013). Drought conditions also impair the stomatal regulatory capability of plant leaves, reduce the synthesis and activity of photosynthetic pigments, and damage the adenosine triphosphate (ATP) photosynthetic apparatus – key factors in diminishing photosynthesis (Zahra et al., 2023). To mitigate oxidative stress under drought conditions, plant cells synthesize superoxide dismutase (SOD), peroxidase (POD), and catalase (CAT). This self-defense system, which includes CAT, ascorbate peroxidase (APX), glutathione (GSH), ascorbate (AsA), among others, plays a pivotal role in this process (Sairam et al., 2011).The application of exogenous plant hormones, including melatonin (MT) and gibberellin (GA), has been shown to beneficially affect the physiological processes of plants subjected to drought stress (Cui et al., 2017; Kamran et al., 2018).

Melatonin, often referred to as the pineal hormone, serves as a crucial antioxidant. It augments the cell’s antioxidant capacity by neutralizing excess reactive oxygen species (ROS) and also affects mitochondrial antioxidant phosphorylation (Zhang et al., 2021). Notably, melatonin not only enhances seed germination but also decelerates cellular aging and enhances stress resistance (Zhang et al., 2015). During episodes of abiotic stress in plants, such as drought, extreme temperatures, or salt alkali conditions, melatonin can directly offer electrons to counteract ROS. Moreover, it influences the activity of certain oxidation and antioxidant enzymes within plants, aiding their growth under adverse conditions (Murch et al., 2009). Although melatonin bolsters plant drought tolerance, a more detailed examination of its specific effects and the underlying mechanisms on millet varieties’ drought resistance is warranted.

In ancient China, millet held precedence among the “five grains” and has since been regarded as a staple crop. Notable for its wide adaptability, drought resistance, and barren tolerance, millet is a critical component of China’s agricultural production (Diao, 2017). Despite its drought-tolerant nature, millet experiences significant growth, developmental, and yield setbacks under seedling stage drought stress (Zhao et al., 2018). Previous research by Ma et al. (Ma et al., 2021) indicates that the application of exogenous melatonin can stimulate maize root development in drought conditions, reducing oxidative system damage and mitigating photosynthetic inhibition, thereby bolstering the maize’s drought resistance. Comparable findings suggest that melatonin application under drought stress enhances antioxidant enzyme activity, increasing both antioxidant and osmoregulatory substance content in soybean seedling leaves (Zou et al., 2019). Zhang et al. (2014) discovered that *Cucumis sativus* L. plants treated with melatonin exhibited significantly higher seed germination rates and root growth rates under drought stress conditions. In addition, Xu et al. (2024) found that pre-soaking seeds in melatonin significantly improved millet germination and antioxidant activity.

Research on the impact of exogenous melatonin on germination and seedling development in diverse millet varieties, as well as the associated physiological mechanisms, remains sparse. This study employs PEG-6000 to mimic a drought stress environment, using foliar spraying application. The aim is to examine the influence of varying melatonin concentrations on millet germination, seedling growth, photosynthetic traits, antioxidant capacity, and osmotic regulatory substance content. This will contribute to our understanding of millet’s drought resistance and provide a scientific foundation for unraveling the physiological mechanisms by which melatonin enhances millet’s drought resistance.

## 2 Materials and methods

### 2.1 Plant materials

The experiment was carried out from July to November 2024 at the Kaiyuan Campus of Henan University of Science and Technology (34°6´N, 112°5´E). The area is hilly and mountainous in western Henan Province, with a warm temperate continental monsoon climate. In this area, the monsoon strength and time are unstable throughout the year, and the precipitation level is low all year round. Yugu 18, Jigu 38, and Changnong 35 were used as the test varieties, and they are the main cultivars in the western region of Henan Province. These millet varieties were provided by the Genetic Improvement Research Group of Special Crops at Henan University of Science and Technology.

### 2.2 Experiment design

The experiment was carried out in the light room of the college of agriculture at henan university of science and technology. The greenhouse conditions were set to a 16-hour day and an 8-hour night cycle, with day and night temperatures maintained at 25 °C and 20 °C respectively. Light intensity was kept at 400 μmol·m^-2^·s^-1^ and humidity was controlled at 75% ± 5%.

Healthy, uniform and plump millet seeds from the varieties Yugu 18, Jigu 38, and Changnong 35 were selected for this study. These seeds were soaked and disinfected in 5% H_2_O_2_ for a duration of 15 minutes, after which the H_2_O_2_ was rinsed off using deionized water. The cleaned seeds were then submerged in 10 mL of melatonin solution at respective concentrations of 0 (CK), 5 (MT5), 25 (MT25), 50 (MT50), 75 (MT75), 100 (MT100), 150 (MT150), and 200 (MT200) μmol/L. Following a 12-hour incubation period in darkness, the seeds were immediately strained and placed in glass petri dishes lined with double filter paper. Each dish contained one hundred seeds, and the experiment was replicated three times.

The Petri dishes, each containing 100 grains, were cultured in a constant-temperature light incubator set at 25 °C, with an 8-hour light and 16-hour dark cycle. This experiment was replicated three times, yielding a total of 24 treatments. Following germination, the seeds were transferred to seedling bowls with dimensions of 12.0 cm in top diameter, 7.5 cm in bottom diameter, and 5.5 cm in height. The cultivation medium consisted of coconut bran substrate, with one plant per pot. The seeds were then placed in a growth chamber for further cultivation. During the 3-4 leaf stage, drought stress treatment was induced by incorporating polyethylene glycol (PEG-6000) at a concentration of 20%. Subsequent to this treatment, sample analysis was conducted.

### 2.3 Determination index and method

#### 2.3.1 Determination of germination test indexes

Germination potential (GP), germination index (GI) and seedling vitality index (VI) were calculated according to the methods described by Abdul-Baki and Anderson (Abdul-Baki and Anderson, 1973). The formulas of these indexes are shown in Xiao et al. (2019):


$$GP=\frac{Number\;of\;germinated\;seeds\;at\;a\;specific\;early\;stage}{Total\;number\;of\;seeds}\times 100\%$$

Here, the specific early stage was determined as the 3rd day after sowing (72 hours), at which the number of initially germinated seeds was counted.


$$GI=\sum_{i=1}^{n}\frac{Gi}{Di}$$

where *G_i_* is the number of germinated seeds on the *i*- th day, and *D_i_* is the corresponding number of days from the start of the experiment.


VI=S×GI

where *S* is the average seedling length (including root and shoot) measured at two stages: 7 days after germination (for root and bud length during the germination assessment) and 14 days after potting (for root and shoot length during post-stress growth evaluation under drought conditions).

#### 2.3.2 Determination of photosynthetic parameters

Three plants exhibiting consistent and complete growth were selected from each treatment group for analysis. The photosynthetic rate (*Pn*) and stomatal conductance (*Gs*) of the first fully-developed leaf were measured using a portable photosynthesis analyzer (Li-6400, LICOR Inc, USA). The instrument was calibrated to measure under the following conditions: The environmental conditions in the measurement area were maintained at a constant temperature of 25°C, a relative humidity of 75% ± 5%, and a light intensity of 400 μmol·m^−^²·s^−^¹, which aligned with the overall greenhouse conditions. Multiple readings were taken from various parts of the leaf, and the average value was recorded.

The intercellular CO_2_ concentration (*Ci)*, transpiration rate (*Tr*), and chlorophyll content were quantified using a SPAD - 502 PLUS portable chlorophyll content meter. These measurements were conducted on the identical leaf as the *Pn* and *Gs* assessments. For each parameter, a minimum of three replicate measurements was obtained, with the mean value subsequently used for analysis.

#### 2.3.3 Determination of physiological and biochemical indexes

Soluble protein content was measured following the Coomassie bright blue G-250 staining method (Nakano and Asada, 1981). About 0.5 g fresh tissue was grinding in liquid nitrogen. Then 5 mL extraction buffer (0.1 M Tris-HCl (pH 7.5), 0.1 M KCl, 1 mM EDTA, 1 mM DTT) was added to the powder and the mixture was vortexed for 30 min at 4°C. After centrifugation at 12,000 rpm for 15 min at 4°C, the supernatant was collected carefully. Take 0.1 mL of supernatant and mix with 5 mL of Coomassie bright blue G-250 reagent. Incubate the solution at room temperature for 5 min and then measure the absorbance of the solution at 595 nm with a spectrophotometer. The content of soluble protein was calculated according to the standard curve of bovine serum albumin (BSA).

Malondialdehyde (MDA) Content was estimated using the thiobarbituric acid (TBA) method (Zhang et al., 2009). 0.5g of plant tissue was homogenized in 5mL of 10% trichloroacetic acid (TCA) solution. The homogenate was centrifuged at 10,000 rpm for 10min at 4°C. 2mL of supernatant was mixed with 2mL of 0.6% TBA prepared in 10% TCA. The mixture was heated in boiling water bath for 30min and then quickly cooled in an ice-water bath. The mixture was further centrifuged at 10,000 rpm for 10min at 4°C and absorbance of the supernatant was measured at 532 nm and 600 nm. MDA content was estimated using the formula given below:


$$MDA\;(\mu mol/g)=\frac{E\times V\times (\text{A}532-\text{A}600)}{W}$$

where A_532_ and A_600_ are the absorbance values at 532 nm and 600 nm respectively, *V* is the volume of the reaction mixture (4 mL), ϵ is the extinction coefficient of MDA (155 mM^−^¹·cm^−^¹), and *W* is the fresh weight of the plant tissue (g).

Estimation of AsA content was performed according to a modified method (Zhang and Kirkham, 1996). Briefly, 0.5 g fresh plant tissue was extracted with 5 mL of 5% metaphosphoric acid. The homogenate was centrifuged at 10,000 rpm for 10 min at 4°C. To 1 mL of the supernatant, 1 mL of 10% trichloroacetic acid, 1 mL of 0.5% 2, 2-dipyridyl in 70% ethanol, and 1 mL of 3% FeCl_3_ were added. The mixture was incubated at 37°C for 40 min and absorbance was measured at 525 nm. The AsA content was determined using the standard curve of pure AsA.

GSH Content was assayed using the method of Guri (Guri, 1983). 0.5g of plant tissue was homogenized in 5mL of 5% sulfosalicylic acid. The homogenate was centrifuged at 10,000 rpm for 10 min at 4°C. 1 mL of supernatant was added to 4 mL of reaction buffer (0.1 M phosphate buffer pH 7.5 containing 0.5 mM EDTA and 0.1 mM 5,5 - dithiobis - (2 - nitrobenzoic acid) (DTNB). The absorbance was measured at 412 nm within five minutes. The GSH content was determined according to the standard curve of reduced glutathione.

Superoxide Dismutase (SOD) activity was assessed using the nitro-blue tetrazolium (NBT) photochemical reduction method (Wang and Huang, 2015). The reaction mixture, which was exposed to fluorescent light for a duration of 15 minutes, comprised 50 mM phosphate buffer (pH 7.8), 13 mM methionine, 75 μM NBT, 10 μM riboflavin, and an enzyme extract derived from 0.5 g of plant tissue as described previously. The absorbance of the resultant mixture was measured at 560 nm. SOD activity was quantified as one unit, which represents the quantity of enzyme required to inhibit the reduction of NBT by 50%.

Peroxidase (POD) activity was determined using the guaiacol method (Fleta-Soriano et al., 2017). The reaction mixture contained 50 mM phosphate buffer (pH 6.0), 20 mM guaiacol, 10 mM H_2_O_2_ and the enzyme extract. The rise in absorbance at 470 nm was measured every 30 s for 3 min. POD activity was expressed as change in absorbance min^− 1^· g^− 1^ of fresh weight.

Catalase activity (CAT) was assayed using the H_2_O_2_ REDOX method (Fleta-Soriano et al., 2017). The reaction mixture contained 50 mM phosphate buffer (pH 7.0), 10 mM H_2_O_2_ and the enzyme extract. The decrease in absorbance at 240 nm was monitored for 3 min, and CAT activity was expressed as amount of H_2_O_2_ decomposed per min per gram of fresh weight.

### 2.4 Data processing and analysis

Data processing was executed utilizing Microsoft Excel 2016, while data analysis was conducted through SPSS 19 software. The Pearson method was adopted for correlation analysis among the indicators. Additionally, mapping was completed using Origin 2021 software.

The calculation of the allelopathic response index (RI) was executed using the formula suggested by Bruce and Richardson (Bruce and Richardson, 1988):


RI=1−C/T (if T≥C)

RI=T/C−1 (if T<C)

In this context, ‘C’ denotes the mean value of the control group, while ‘T’ signifies the mean value of the treatment group. A positive allelopathic effect on the growth of the evaluated plants is indicated by RI>0. Conversely, a negative allelopathic effect is suggested when RI<0. The intensity of the allelopathic effect can be measured by the absolute value of RI (|RI|).

The Theoretical effect of allelopathy (SE) is defined as the mean value of the allelopathy response index (RI) across all measurement indices. A positive SE value (SE>0) suggests a theoretical allelopathic promotion, while a negative SE value (SE<0) implies a theoretical allelopathic inhibition. The magnitude of the allelopathic effect is represented by the absolute value of SE (|SE|). The formula for calculation is provided below:


$$SE=\frac{RI1+RI2+RI3+\cdots \cdots +RIn}{n}$$

When n is the number of measured indices, and RIi is the allelopathic response index for the i-th measured index.

Membership function value Equation 1:


(1) R(Xi)=(Xi−Xmin)/(Xmax−Xmin)

Inverse membership function value Equation 2:


(2) R(Xi)=1−(Xi−Xmin)/(Xmax−Xmin)

Where: Xi is the measured value of the index; Xmax and Xmin are the maximum and minimum values of the processing indexes, respectively. R(Xi) represents the membership function value of the indicator Xi, and then the membership function value of each treatment is summed to determine the average value. The average membership function is used to assess the drought resistance of different treatments. A higher value indicates stronger drought resistance ability. If the measured index is positively correlated with drought stress, the membership function (1) is applied, and if it is negatively correlated, the inverse membership function (2) is applied.

## 3 Results

### 3.1 Effects of different concentrations of melatonin on seed germination of millet

The germination indices of millet varieties subjected to varying melatonin concentrations exhibited an initial increase followed by a decrease (**Table 1**). For melatonin concentrations ranging from 5-100 μmol·L^-1^, the germination rate, germination potential, germination index, and vitality index for Yugu 18 and Jigu 38 seeds consistently rose with increasing solution concentration. The MT100 treatment achieved the peak values, marking significant increments of 21.43%, 50.93%, 46.22%, and 47.54% for Yugu 18, and 71.60%, 71.34%, 55.64%, and 71.34% for Jigu 38 compared to the control. Meanwhile, for melatonin concentrations between 5-125 μmol·L^-1^, similar indices for changnong 35 seeds also showed an upward trend with higher solution concentrations. The MT125 treatment reached a zenith, registering increases of 69.22%, 71.91%, 53.47%, and 58.16% relative to the control.

**Table 1:** Effects of different concentrations of melatonin on seed germination in millet varieties.

Variety	Content	Determination index				Allelopathy index				
		GR(%)	GP(%)	GI(%)	VI(%)	GR	GP	GI	VI	SE
Yugu 18	CK	23.33 ± 2.67c	17.67 ± 1.45c	15.99 ± 2.35b	49.28 ± 4.93c					
	MT_5_	22.67 ± 0.66b	19.33 ± 0.88c	16.57 ± 1.29b	49.67 ± 12.13c	-0.03	0.09	0.04	0.01	0.03
	MT_25_	24.33 ± 1.20b	22.67 ± 0.88b	18.14 ± 0.50b	48.41 ± 2.03c	0.04	0.15	0.12	-0.02	0.07
	MT_50_	25.67 ± 0.33b	23.67 ± 0.67b	18.99 ± 2.23b	55.25 ± 1.87b	0.09	0.04	0.16	0.11	0.10
	MT_75_	26.33 ± 0.33c	24.67 ± 0.33b	22.42 ± 3.53b	65.48 ± 2.02c	0.11	0.01	0.29	0.25	0.17
	MT_100_	28.33 ± 0.67c	26.67 ± 0.88c	23.38 ± 1.16b	72.71 ± 12.74b	0.18	0.10	0.32	0.32	0.23
	MT_125_	25.67 ± 0.67c	23.33 ± 0.88c	21.00 ± 3.51c	69.61 ± 4.90c	0.09	0.14	0.24	0.29	0.19
	MT_150_	24.33 ± 2.03c	20.67 ± 1.52c	14.14 ± 3.45c	43.11 ± 11.18b	0.04	0.13	-0.13	-0.14	-0.03
	MT_200_	22.33 ± 1.45c	18.67 ± 1.20c	10.85 ± 1.50c	30.43 ± 5.18b	-0.04	0.11	-0.47	-0.62	-0.26
Jigu 38	CK	31.66 ± 1.20b	27.67 ± 2.40b	24.28 ± 2.86b	75.19 ± 0.96b					
	MT_5_	41.67 ± 2.03a	29.33 ± 4.26a	25.95 ± 1.58b	77.06 ± 1.38b	0.24	0.06	0.06	0.02	0.10
	MT_25_	42.33 ± 2.33a	33.67 ± 1.67a	31.04 ± 0.33b	81.21 ± 10.51b	0.25	0.18	0.22	0.07	0.18
	MT_50_	45.67 ± 4.41a	40.67 ± 2.40a	33.52 ± 1.53a	87.89 ± 4.81ab	0.31	0.32	0.28	0.14	0.26
	MT_75_	46.33 ± 3.28b	44.33 ± 2.33b	35.62 ± 0.96a	106.23 ± 0.61b	0.32	0.38	0.32	0.29	0.33
	MT_100_	54.33 ± 3.38b	47.33 ± 3.18a	37.76 ± 0.81b	128.83 ± 4.82a	0.42	0.42	0.36	0.42	0.41
	MT_125_	41.67 ± 3.17b	41.67 ± 0.88b	33.61 ± 1.77b	116.94 ± 4.20b	0.24	0.34	0.28	0.36	0.31
	MT_150_	40.33 ± 0.88b	40.33 ± 0.88b	31.76 ± 1.12b	118.90 ± 5.34a	0.21	0.31	0.24	0.37	0.28
	MT_200_	38.33 ± 3.48b	38.33 ± 1.20b	27.14 ± 1.44b	57.62 ± 4.83b	0.17	0.28	0.11	-0.30	0.07
Changnong 35	CK	47.67 ± 1.21d	40.33 ± 0.67a	33.14 ± 1.78a	103.65 ± 8.66a					
	MT_5_	39.33 ± 1.76a	35.67 ± 1.20a	33.95 ± 0.70a	104.63 ± 1.91a	-0.21	-0.13	0.02	0.01	-0.08
	MT_25_	40.67 ± 2.33a	36.67 ± 2.73a	36.14 ± 1.36a	121.82 ± 7.62a	-0.17	-0.10	0.08	0.15	-0.01
	MT_50_	42.33 ± 1.76a	39.67 ± 0.88a	38.05 ± 1.27a	125.52 ± 3.34a	-0.13	-0.02	0.13	0.17	0.04
	MT_75_	60.33 ± 3.18a	49.33 ± 0.88a	42.62 ± 2.19a	131.69 ± 3.72a	0.21	0.18	0.22	0.21	0.21
	MT_100_	62.67 ± 1.20a	53.33 ± 1.85a	45.95 ± 1.68a	139.70 ± 6.23a	0.24	0.24	0.28	0.26	0.26
	MT_125_	80.67 ± 2.19a	69.33 ± 1.45a	50.86 ± 2.41a	163.93 ± 9.76a	0.41	0.42	0.35	0.37	0.39
	MT_150_	63.33 ± 1.20a	50.33 ± 0.66a	48.38 ± 7.37b	154.55 ± 14.15a	0.25	0.20	0.32	0.33	0.28
	MT_200_	55.67 ± 0.88a	45.67 ± 1.20a	44.61 ± 2.97a	133.15 ± 11.34a	-0.14	-0.12	0.26	0.22	0.06

GR, Germination rate; GP, Germination potential; GI, Germination index; VI, Vitality index; Vitality Index; Different lowercase letters in the same column indicate significant differences (*P*<0.05). Different lowercase letters in the same column indicate significant differences between different varieties and concentrations (*P*<0.05).

When the concentration exceeded MT125, a significant decrease in the germination indices of different millet cultivars was observed. The magnitude of this decrease was directly proportional to the solution concentration. At a concentration of MT200, the Yugu 18 treatment resulted in reductions in germination rate, germination index, and vitality index by 4.29%, 32.15%, and 39.25% respectively, relative to the control (CK). Simultaneously, the vitality indices for the Changnong 35 and Jigu 38 treatments significantly decreased by 23.27% and 22.16% respectively. These results indicate that melatonin treatment exacerbates the negative effects on millet germination to a certain extent. Different concentrations had varying effects on the germination of different cultivars, with higher concentrations inhibiting seed germination.

The comprehensive allelopathic effect index provides insights into both the autotoxic effect and the influence of melatonin concentration on seed germination. As the solution concentration varied between 5 and 200 μmol·L^-1^, distinct melatonin concentrations first enhanced and then suppressed the germination of millet varieties. The inhibitory impact of melatonin on millet seed germination became more pronounced at concentrations of MT150 and MT200. At equivalent inhibitory concentrations, the suppressive effect of melatonin was most pronounced for Yugu 18, while it was decreased intensity for Jigu 38 and Changnong 35.

### 3.2 Effects of different concentrations of melatonin on millet growth indexes under drought stress

Compared to the control (CK), melatonin’s varying concentrations exhibited differential effects on the germination bud length, root length of millet varieties, seedling length, and root length under drought stress (**Table 2**). During seed germination, Yugu 18 and Jigu 38 showed an initial decrease in bud length and root length, subsequently increasing, with both attaining their maximum value at MT100. These increases were 14.73% and 71.14% for bud length, and 84.03% and 27.39% for root length, respectively, compared to the control. For Changnong 35, the maximum bud length was achieved at MT150, and the maximum root length at MT125, representing increases of 6.06% and 2.97%, respectively, relative to the control. However, when the solution concentration surpassed MT125, the germination indices of different millet varieties commenced to decrease, with the intensity of decrease escalating as the solution concentration increased. Notably, the bud length of Yugu 18 decreased by a mere 9.01%.

**Table 2:** Effects of different concentrations of melatonin on bud length, seedling length and root length of millet under drought stress.

Variety	Content	Germination index		Pot index	
		BG	RL	SL	RL
Yugu 18	CK	30.24 ± 2.06a	49.20 ± 2.72b	138.61 ± 6.45a	124.21 ± 2.22a
	MT_5_	29.40 ± 9.33a	49.09 ± 4.21b	117.62 ± 5.44a	98.61 ± 3.53a
	MT_25_	26.68 ± 1.38a	61.84 ± 5.38a	107.35 ± 4.75a	148.14 ± 3.66a
	MT_50_	19.67 ± 5.12a	74.54 ± 1.36a	127.97 ± 5.89a	157.56 ± 6.22a
	MT_75_	24.52 ± 1.57a	79.88 ± 4.06a	139.37 ± 7.22a	171.81 ± 5.34a
	MT_100_	34.70 ± 8.58a	84.83 ± 2.23a	165.05 ± 3.70a	191.14 ± 3.42a
	MT_125_	31.82 ± 7.44a	59.58 ± 4.82b	148.38 ± 5.87ab	175.44 ± 9.01a
	MT_150_	30.47 ± 4.29a	74.91 ± 3.95a	139.38 ± 7.38a	185.30 ± 0.62a
	MT_200_	27.74 ± 1.86a	49.65 ± 2.78b	144.25 ± 7.56a	172.92 ± 6.69a
Jigu 38	CK	21.42 ± 2.88b	68.83 ± 3.69a	124.47 ± 2.67b	152.07 ± 5.01a
	MT_5_	18.70 ± 2.10a	74.72 ± 4.82a	92.67 ± 10.30b	141.26 ± 4.40b
	MT_25_	23.93 ± 6.53a	62.42 ± 6.61a	117.31 ± 8.61a	137.50 ± 3.11a
	MT_50_	25.93 ± 8.84a	65.45 ± 5.11b	134.53 ± 9.30b	153.37 ± 3.45a
	MT_75_	32.63 ± 4.74b	85.15 ± 3.36a	135.40 ± 3.82a	165.57 ± 3.03a
	MT_100_	39.42 ± 1.67a	87.68 ± 3.92a	145.58 ± 5.13a	185.60 ± 0.04a
	MT_125_	35.05 ± 4.85a	86.40 ± 5.01a	127.15 ± 4.39c	174.03 ± 6.77a
	MT_150_	37.47 ± 2.62a	65.80 ± 2.31a	118.09 ± 4.93b	161.48 ± 7.66a
	MT_200_	34.43 ± 2.19a	84.44 ± 4.43b	121.05 ± 4.25b	176.28 ± 4.80a
Changnong 35	CK	31.17 ± 1.71a	47.49 ± 5.14b	93.54 ± 5.83c	156.25 ± 0.07a
	MT_5_	29.71 ± 2.87a	40.49 ± 2.72c	86.26 ± 8.79b	140.73 ± 2.76b
	MT_25_	25.47 ± 1.15a	30.48 ± 5.57a	120.33 ± 8.54a	129.09 ± 6.20a
	MT_50_	22.60 ± 1.00a	36.12 ± 4.56c	132.49 ± 5.46a	143.16 ± 1.26a
	MT_75_	20.16 ± 0.45a	34.08 ± 7.60b	138.65 ± 4.97a	152.96 ± 5.54a
	MT_100_	30.26 ± 4.45a	42.67 ± 3.67b	143.29 ± 8.48b	163.81 ± 1.08a
	MT_125_	33.06 ± 1.68a	48.92 ± 4.49c	154.71 ± 10.69a	205.98 ± 1.27a
	MT_150_	32.04 ± 0.71a	46.37 ± 6.49b	97.62 ± 3.72c	183.29 ± 4.08a
	MT_200_	29.79 ± 1.61a	42.96 ± 5.49a	119.03 ± 5.98b	170.31 ± 3.82a

BG, Bud growth; SL, Seedling length; RL, Root length; Different lowercase letters in the same column indicate significant differences (P< 0.05). Different lowercase letters in the same column indicate significant differences between different varieties and concentrations (P< 0.05).

Under drought stress conditions, millet seedling length and root length exhibited an initial decrease followed by an increase (**Table 2**). Specifically, the seedling length and root length of Yugu 18 and Jigu 38 reached their peak values at MT100, showing increases of 19.07% and 53.88%, and 16.95% and 22.05%, respectively, when compared to the control group. In contrast, Changnong 35 achieved its maximum seedling length and root length at MT125, with increases of 65.39% and 31.83%, respectively, relative to the control. These results suggest that melatonin treatment can enhance millet germination and growth to a certain degree. However, it is important to note that different concentrations of melatonin yielded varying effects on millet growth, with higher concentrations acting as a growth suppressant.

### 3.3 Effects of different concentrations of melatonin on photosynthetic parameters of millet under drought stress

**Figure 1:**
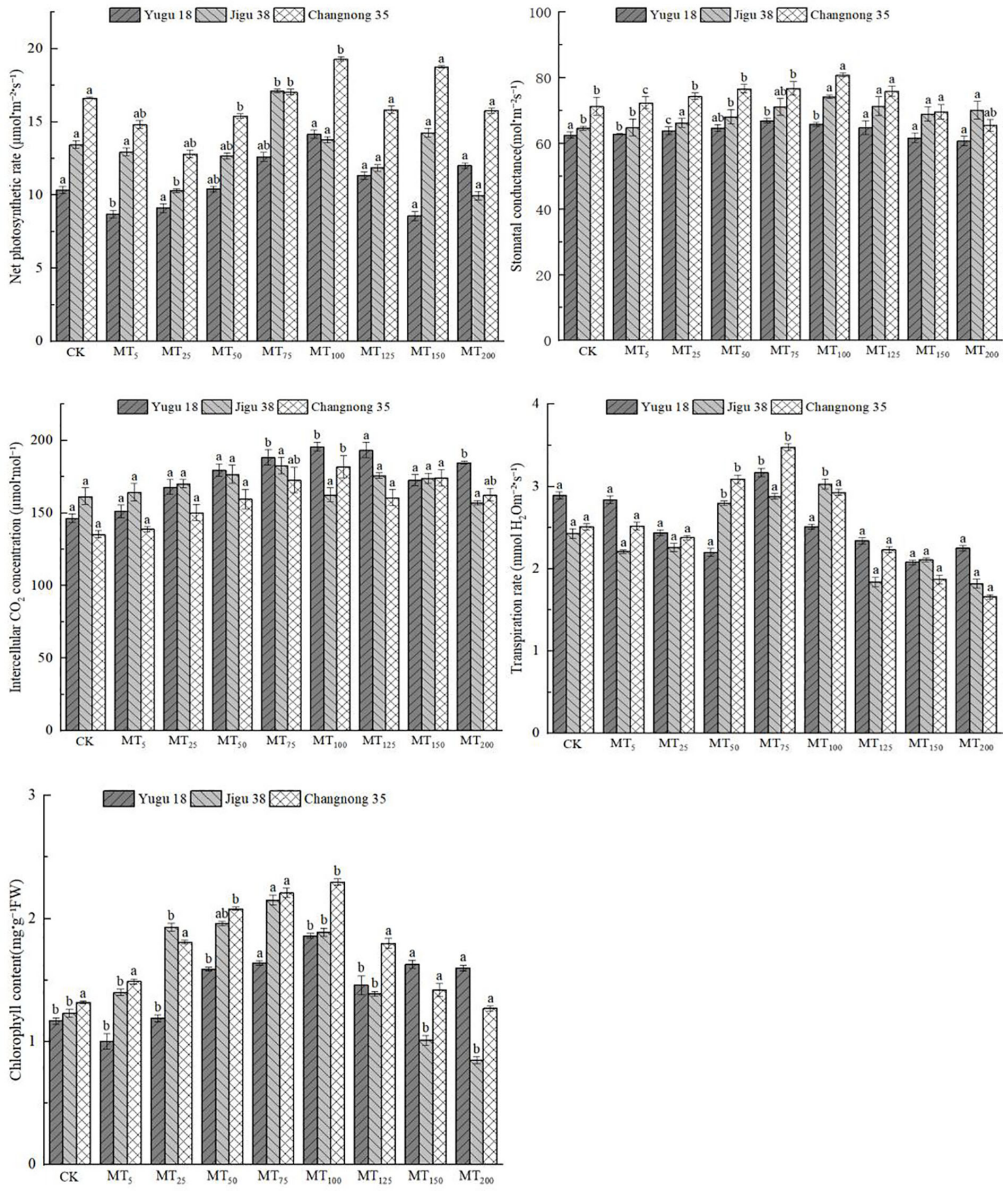
Effects of different concentrations of melatonin on photosynthetic parameters of millet seedlings under drought stress. Different lowercase letters indicate significant differences between treatments at P < 0.05 (one-way ANOVA, Tukey’s test).

The rate of photosynthesis provides a direct indication of the growth state of plants under drought stress conditions. This study observed the varying impacts of drought stress on several parameters, including the net photosynthetic rate (Pn), stomatal conductance (Gs), intercellular CO_2_ concentration (Ci), transpiration rate (Tr), and chlorophyll content (CC) in millet seedlings of distinct varieties (**Figure 1**). The highest Pn values for Yugu 18 and Changnong 35 were recorded at MT100, while Jigu 38 peaked at MT75. In comparison to the control group, Pn increased by 36.98%, 16.12%, and 27.21% respectively. However, a marked decrease in Pn was noted with increasing concentration. Yugu 18 and Jigu 38 recorded their lowest Pn levels under MT150 and MT200 treatments, indicating decreases of 16.94% and 25.95% respectively when compared to the control. For Changnong 35, the nadir was observed only under the MT25 treatment, registering a decrease of 29.82% relative to the control.

The Gs of Yugu 18, Jigu 38, and Changnong 35 exhibited an initial increase followed by a decrease. Both Yugu 18 and Jigu 38 peaked under the MT75 treatment. In contrast, Jigu 38 and Changnong 35 achieved their maximum germination rates with the MT100 treatment, marking increases of 6.89%, 14.87%, and 13.46% respectively, in comparison to the control group. As the concentration increased, Yugu 18 and Changnong 35 experienced their lowest germination rates under the MT200 treatment, with reductions of 2.82% and 8.19% respectively, when compared to the control.

The Ci of Yugu 18 and Changnong 35 initially increased, then decreased. For both Yugu 18 and Changnong 35, their Ci peaked under the MT100 treatment. In contrast, Jigu 38 reached its maximum Ci under the MT75 treatment, marking an increase of 33.96%, 34.46%, and 13.15% respectively when compared to the control. As the concentration increased, Jigu 38’s Ci diminished to its lowest level under the MT200 treatment, which was 2.75% less than the control.

The Ci of Yugu 18 and Changnong 35 initially increased, then decreased. For both Yugu 18 and Changnong 35, their Ci peaked under the MT100 treatment. In contrast, Jigu 38 reached its maximum Ci under the MT75 treatment, marking an increase of 33.96%, 34.46%, and 13.15% respectively when compared to the control. As the concentration increased, Jigu 38’s Ci diminished to its lowest level under the MT200 treatment, which was 2.75% less than the control.

The Tr of millet varieties initially declined before subsequently increasing. Specifically, the Tr of Yugu 18 and Changnong 35 reached their peak levels under the MT75 treatment, while Jigu 38 achieved its highest level under the MT100 treatment. These represented increases of 9.69%, 38.65%, and 24.69% respectively, compared to the control group. However, as the concentration increased, both Jigu 38 and Changnong 35 decreased to their lowest levels under the MT200 treatment, while Yugu 18 reached its minimum under the MT150 treatment. These decreases amounted to 25.10%, 33.86%, and 28.03% respectively, when compared to the control group.

The CC of millet varieties initially increased before subsequently decreasing. Yugu 18 and Changnong 35 achieved their peak values under the MT100 treatment, while Jigu 38 reached its maximum value under the MT75 treatment. These represented increases of 58.97%, 73.48%, and 74.79% respectively, when compared to the control group. As the concentration increased, the rate of decrease became more significant, with Yugu 18 and Changnong 35 reaching their minimum values of 30.89% and 24.31% respectively, under the MT200 treatment.

### 3.4 Effects of different concentrations of melatonin on MDA content in millet seedlings under drought stress

**Figure 2:**
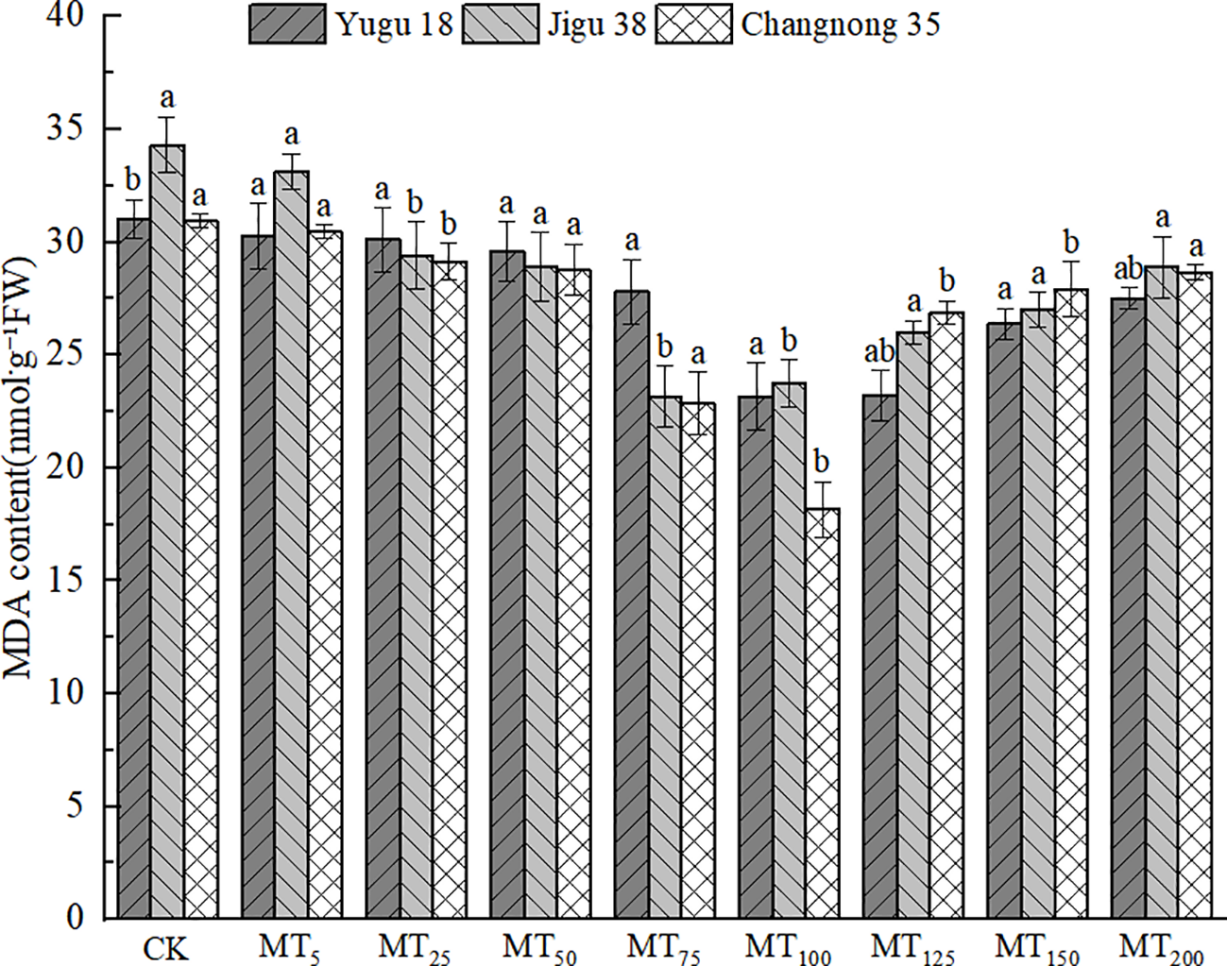
Effects of different concentrations of melatonin on MDA content of millet seedlings under drought stress. Different lowercase letters indicate significant differences between treatments at P < 0.05 (one-way ANOVA, Tukey’s test).

Under drought stress, the MDA content in millet seedling tissues significantly increased under the CK treatment (**Figure 2**). However, following melatonin treatment, there was an initial trend of MDA content reduction compared to the CK treatment. Specifically, the MDA content of Yugu 18 and Changnong 35 decreased to their lowest levels under the MT100 treatment, with significant reductions of 25.31% and 41.29% respectively, compared to the CK treatment. For Jigu 38, its MDA content decreased to its lowest under the MT75 treatment, with a significant reduction of 32.61% compared to the CK treatment.

### 3.5 Effects of different concentrations of melatonin treatment on soluble protein content in millet seedlings under drought stress

**Figure 3:**
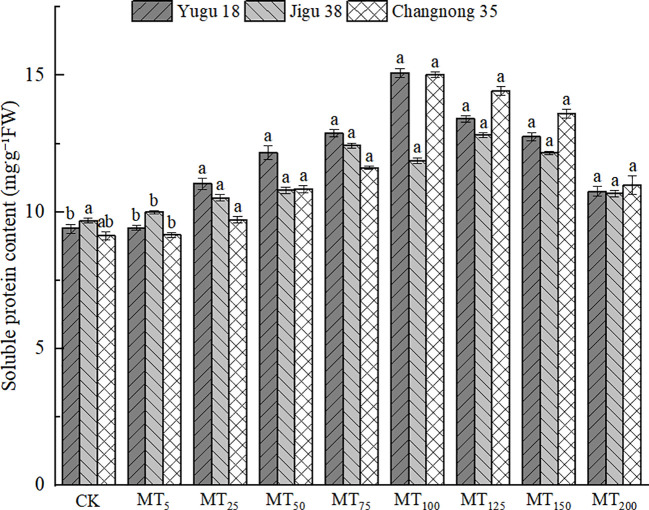
Effects of different concentrations of melatonin on soluble protein content of millet seedlings under drought stress. Different lowercase letters indicate significant differences between treatments at P < 0.05 (one-way ANOVA, Tukey’s test).

Drought stress led to a significant increase in soluble protein content when treated with exogenous melatonin (**Figure 3**). The maximum soluble protein content for Yugu 18 and Changnong 35 was observed under MT100 treatment, registering increases of 60.74% and 64.68% respectively, compared to the CK treatment. For Jigu 38, the highest soluble protein content was noted under MT125 treatment, marking a 32.36% increase relative to the CK treatment. At a melatonin concentration of MT200, the soluble protein content of Yugu 18, Jigu 38, and Changnong 35 increased by 14.47%, 10.24%, and 20.29% respectively, compared to the CK treatment.

### 3.6 Effects of different concentrations of melatonin on the contents of AsA and GSH in millet seedlings under drought stress

**Figure 4:**
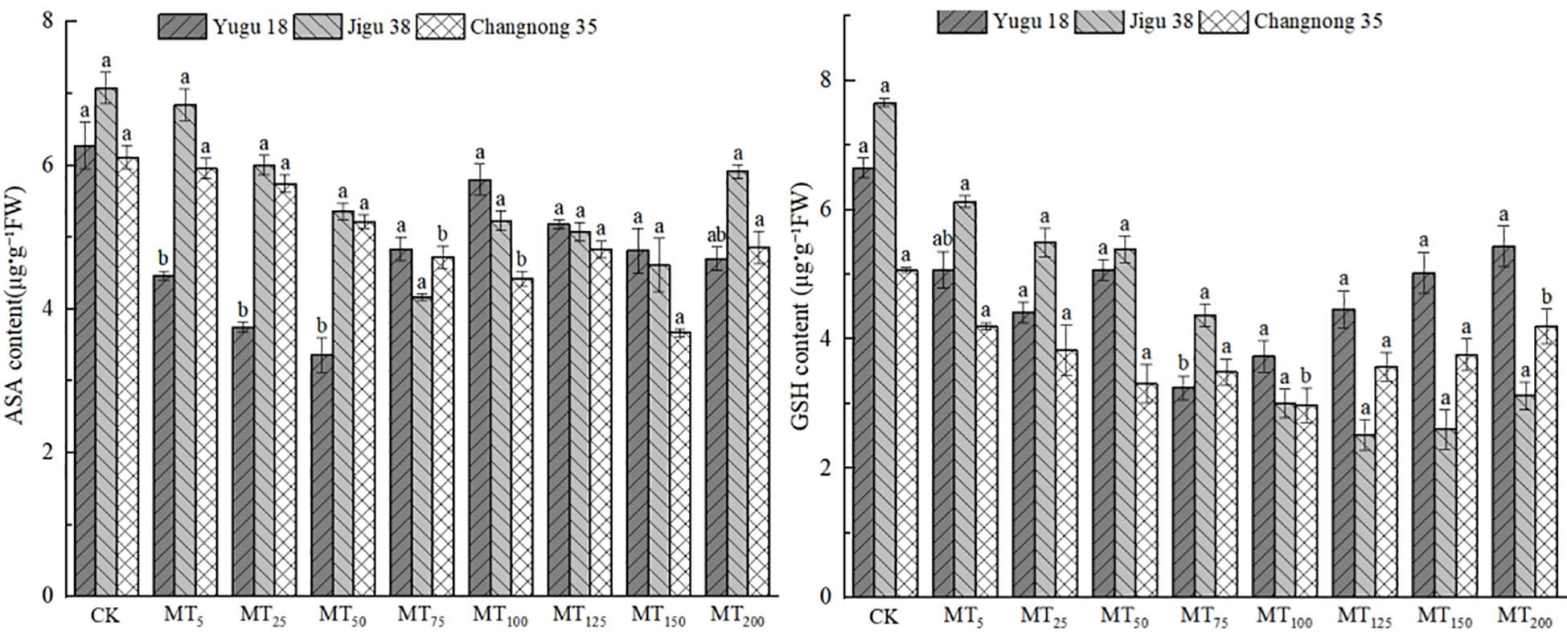
Effects of different concentrations of melatonin on ASA and GSH contents of millet seedlings under drought stress. Different lowercase letters indicate significant differences between treatments at P < 0.05 (one-way ANOVA, Tukey’s test).

ASA and GSH are pivotal in conferring plant resistance to oxidative stress, with the AsA content level being intimately linked to the stress resistance of millet varieties. Under conditions of drought stress, there was a notable reduction in the AsA content in millet seedlings (**Figure 4**). Specifically, the AsA content in Yugu 18, Jigu 38, and Changnong 35 exhibited decreases ranging from 7.51% to 46.50%, 3.32% to 41.92%, and 2.57% to 40.01%, respectively.

The GSH content in millet seedlings exhibited a significant decrease, with reduction rates ranging from 17.21% to 41.38%, 19.97% to 67.13%, and 0.91% to 35.99% when compared to the control treatment for Yugu 18, Jigu 38, and Changnong 35, respectively.

### 3.7 Effects of different concentrations of melatonin treatment on antioxidant oxidase activity of millet seedlings under drought stress

**Figure 5:**
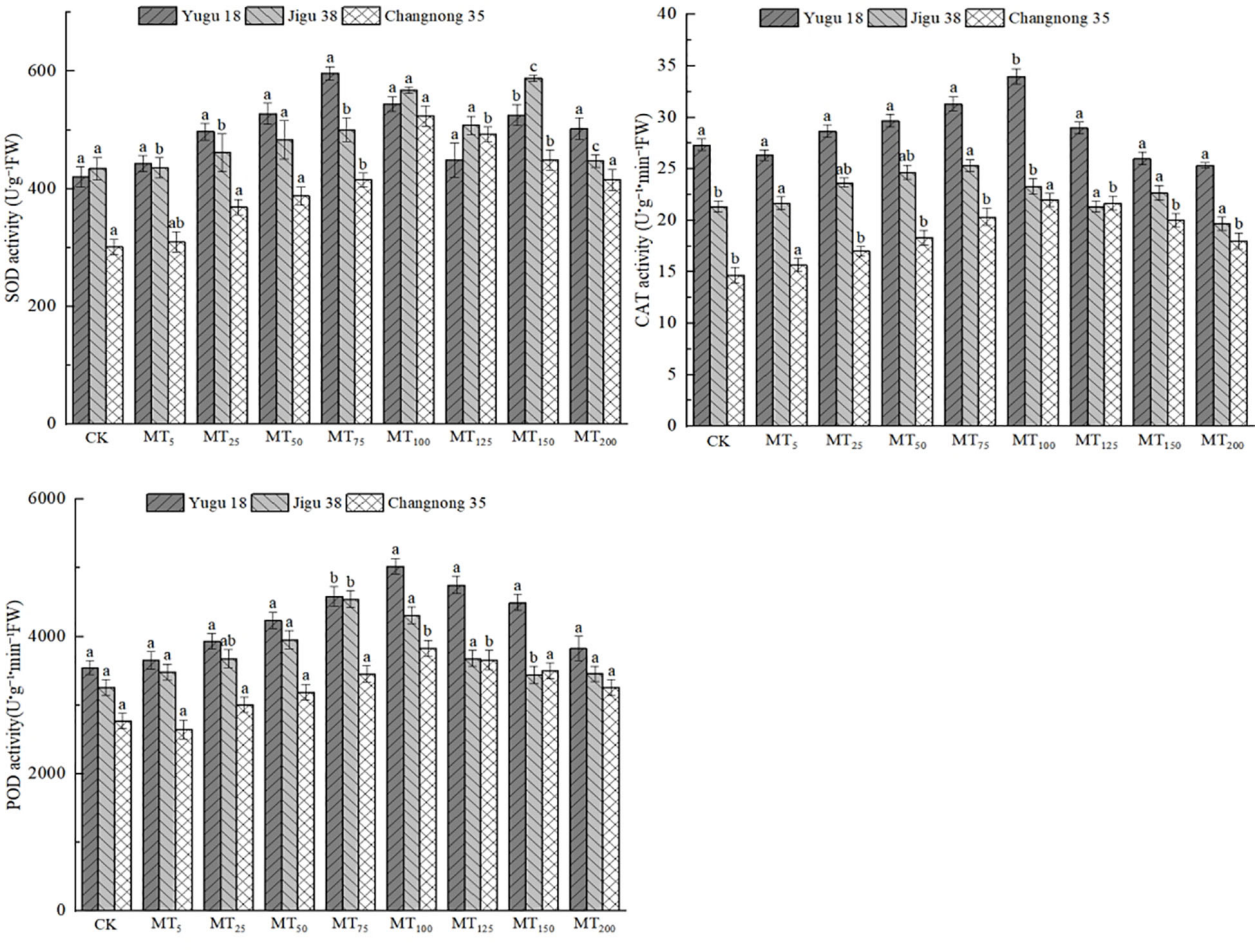
Effects of different concentrations of melatonin on antioxidant oxidase activities of millet seedlings under drought stress. Different lowercase letters indicate significant differences between treatments at P < 0.05 (one-way ANOVA, Tukey’s test).

Under drought stress conditions, the activities of SOD, CAT, and POD in different millet varieties experienced a significant increase (**Figure 5**). The SOD activity of Yugu 18 reached its peak under the MT75 treatment, registering a significant increase of 73.78% compared to the CK treatment. For Jigu 38 and Changnong 35, their highest SOD activities were observed under the MT100 treatment, showing increases of 30.63% and 1.84%, respectively, compared to the CK treatment. Generally, under drought stress, the CAT activity across millet varieties rose notably. Both Yugu 18 and Changnong 35 demonstrated their peak CAT activities under the MT100 treatment, marking increases of 24.41% and 49.96% respectively in comparison to the CK treatment. Meanwhile, Jigu 38 exhibited its highest CAT activity under the MT75 treatment, reflecting an increase of 18.75% compared to the CK treatment. As for POD activity, Yugu 18 and Changnong 35 had their highest levels under the MT100 treatment, with increases of 41.74% and 38.23% respectively relative to the CK treatment. In contrast, Jigu 38 attained its peak POD activity under the MT75 treatment, showcasing a 39.58% increase compared to the CK treatment.

### 3.8 Principal component analysis and correlation analysis of index changes of millet seedlings treated with different melatonin concentrations under drought stress

**Figure 6:**
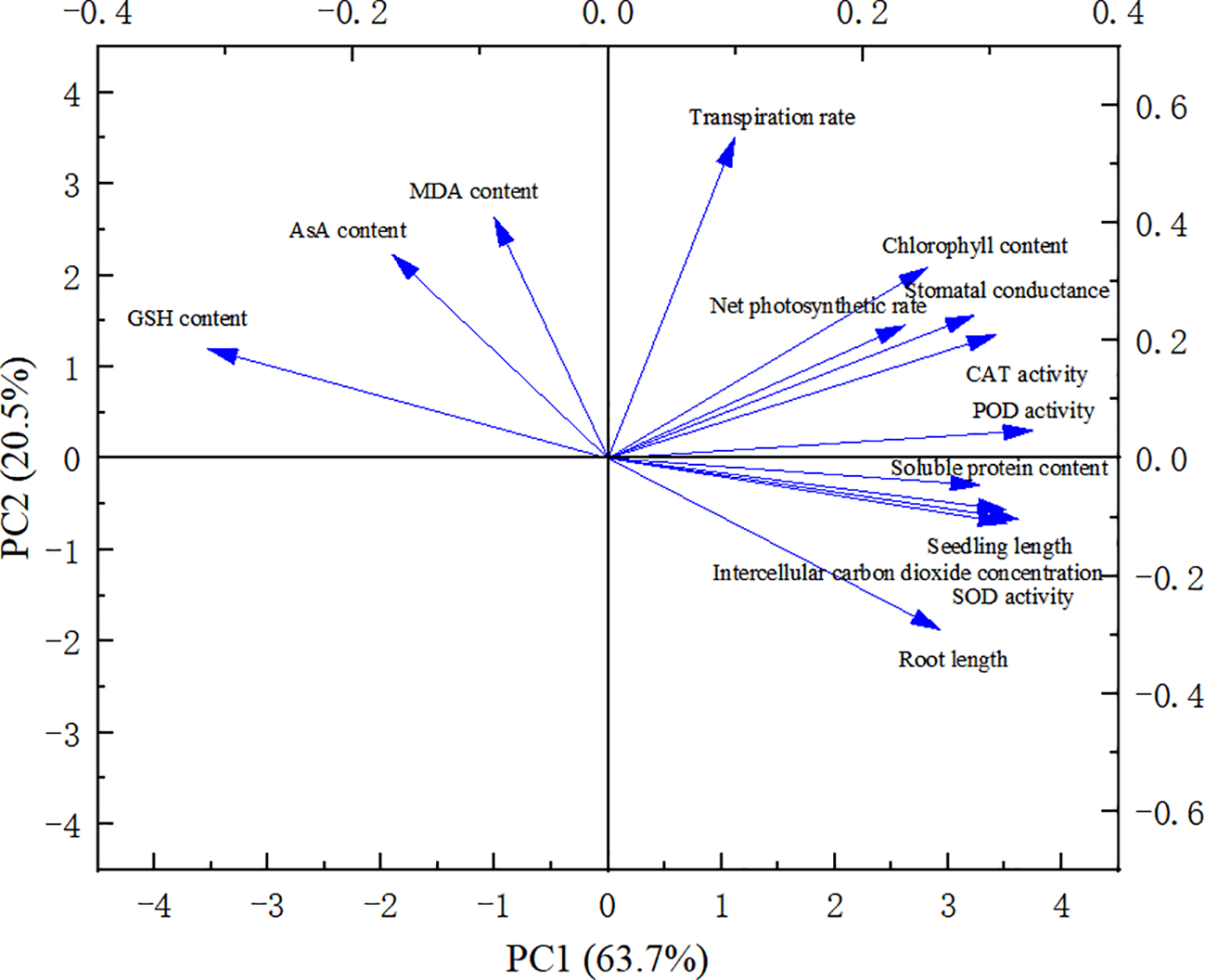
Principal component analysis of each index under drought stress.

A comprehensive evaluation of the changes in millet variety indices under varying concentration treatments necessitates more than a single index to accurately capture the overall performance. Consequently, we conducted a principal component analysis (PCA) on 14 major millet indices (**Figure 6**). The variance contribution rate of the first principal component (PC1) was 63.7%. This component represents factors such as seedling length, root length, photosynthetic rate, stomatal conductance, intercellular CO_2_ concentration, transpiration rate, chlorophyll content, soluble protein content, and the activities of SOD, CAT, and POD. The second principal component (PC2) had a variance contribution rate of 20.5% and was associated with MDA content, AsA content, and GSH content activity.

The indices were normalized, and their respective weights were subsequently computed (**Table 3**). Among the factors assessed, the five most influential were identified as POD activity, Ci, GSH content, soluble protein content, and SOD activity, listed in descending order of significance.

**Table 3:** Component load matrix, eigenvector and weight analysis of millet indexes under drought stress.

Parameter	Principal component 1		Principal component 2		Principal component 3		Weight(%)	Influence order
	Eigenvector	Loading matri(*x*)	Eigenvector	Loading matri(*x*)	Eigenvector	Loading matri(*x*)		
Seedling length	0.291	0.87	-0.045	-0.077	-0.121	-0.122	11.99	7
root length	0.260	0.777	-0.291	-0.493	0.218	0.22	10.71	9
photosynthetic rate	0.233	0.696	0.227	0.384	0.517	0.523	9.59	11
stomatal conductance	0.287	0.858	0.243	0.412	-0.270	-0.273	11.82	8
intercellular CO_2_ concentration	0.322	0.96	-0.103	-0.175	0.029	0.029	13.23	2
transpiration rate	0.099	0.297	0.545	0.924	-0.035	-0.035	4.07	13
chlorophyll content	0.251	0.748	0.324	0.549	-0.317	-0.32	10.30	10
MAD content	-0.089	-0.266	0.410	0.695	0.623	0.63	3.70	14
Soluble protein content	0.313	0.935	-0.110	-0.186	0.063	0.064	12.89	4
ASAcontent	-0.169	-0.505	0.347	0.588	-0.194	-0.196	7.01	12
GSH content	-0.314	-0.937	0.186	0.315	0.002	0.002	12.97	3
SOD activity	0.313	0.933	-0.087	-0.148	0.204	0.206	12.86	5
CAT activity	0.304	0.909	0.210	0.355	-0.154	-0.156	12.53	6
PODactivity	0.333	0.993	0.047	0.08	-0.013	-0.013	13.69	1

**Figure 7:**
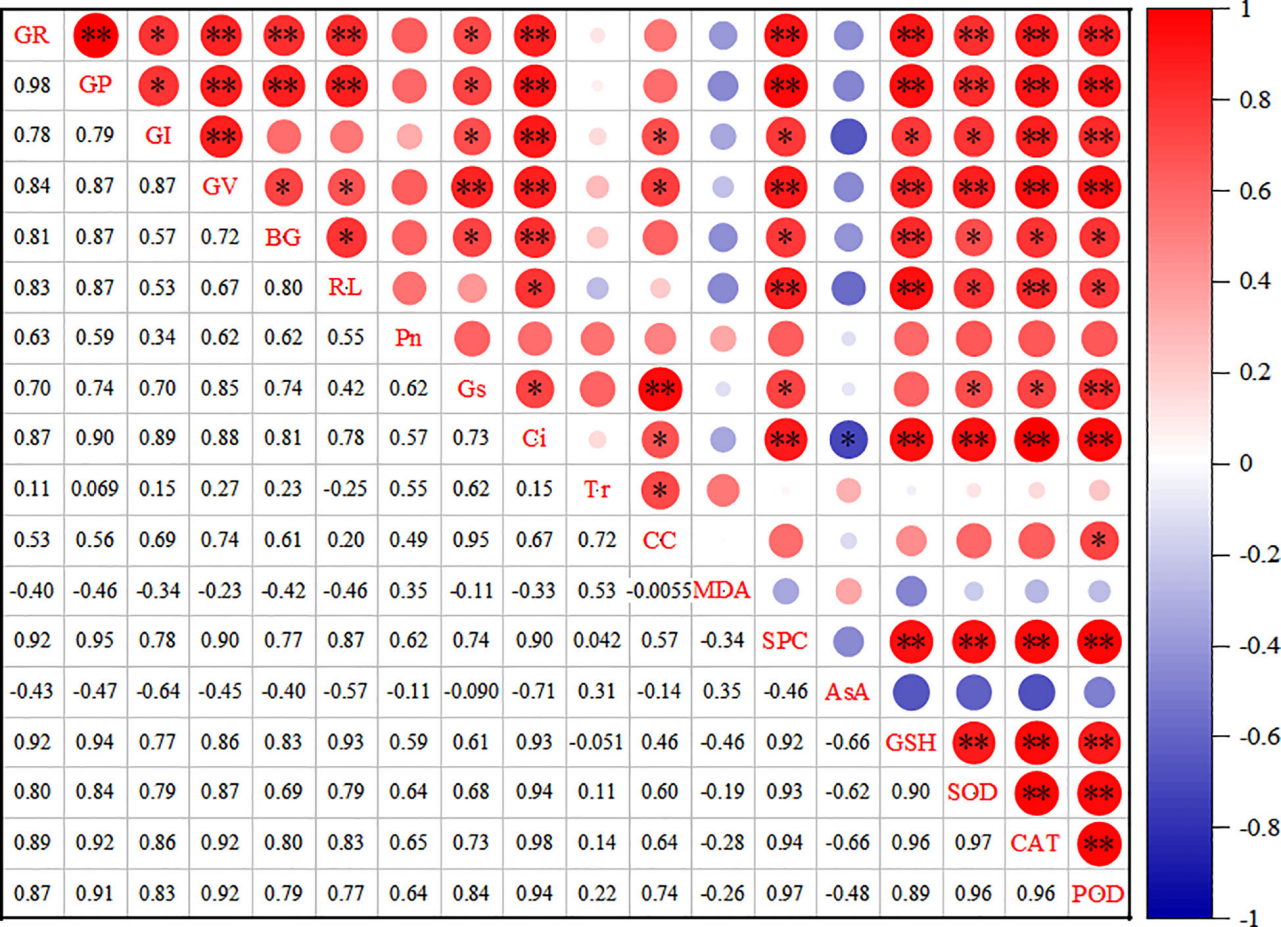
Correlation analysis of each index under drought stress. * indicates statistical significance at P < 0.05, and ** indicates highly significant at P < 0.01.

The correlation analysis results (**Figure 7**) indicate a significant positive relationship between the germination rate of millet varieties and several factors, including germination potential, vigor index, seedling length, root length, Ci, soluble protein,GSH, SOD,CAT, and POD (P< 0.01). Additionally, the germination index and stomatal conductance also positively influenced millet germination (P< 0.05). Furthermore, germination potential showed a significant positive correlation with germination activity, seedling length, root length, Ci, soluble protein, GSH, SOD, CAT, and POD (P< 0.01), as well as with the germination index and stomatal conductance (P< 0.05). Seedling length was significantly positively correlated with intercellular CO_2_ and GSH (P< 0.01). Root length demonstrated a significant positive correlation with itself, stomatal conductance, soluble protein, SOD, CAT, and POD (P< 0.05). Moreover, root length was significantly positively correlated with soluble protein, GSH, and GAT (P< 0.01), as well as with intercellular CO_2_, SOD, and POD (P< 0.05).

### 3.9 Membership function values of physiological indexes of millet seedlings treated with different concentrations of melatonin under drought stress

The average membership function values for the eight drought stress treatments varied significantly, ranging from 0.2852 to 0.8605. The highest average membership function values for AsA content, GSH content, CAT, and POD activity were observed in the MT100 treatment. Conversely, the MT5 treatment exhibited the highest average membership function values for MDA content. The MT125 treatment recorded the highest average membership function values for soluble protein content, while the MT150 treatment showed the highest values for SOD activity. Based on a comprehensive evaluation of these membership function values, the drought resistance of the eight treatments can be ranked as follows: MT100>MT75>MT150>MT125>MT50>CK>MT200>MT25>MT5.This suggests that the MT100 treatment has the strongest drought resistance, whereas the MT5 and MT25 treatments exhibit the weakest resistance (**Table 4**).

**Table 4:** Average membership function values of millet physiological indexes under drought stress.

Treatment	MAD content	Soluble protein content	ASAcontent	GSH content	SOD activity	CAT activity	POD activity	Average value	Rank
CK	0.9349	0.4245	0.6521	0.3113	0.0858	0.2241	0.3134	0.4209	6
MT_5_	0.9584	0.0277	0.5132	0.2741	0.0681	0.1329	0.0218	0.2852	9
MT_25_	0.2840	0.2216	0.4819	0.2819	0.3579	0.3315	0.3273	0.3266	8
MT_50_	0.8231	0.4067	0.2784	0.3844	0.5072	0.5761	0.5318	0.5011	5
MT_75_	0.8099	0.6330	0.2506	0.8576	0.7437	0.9022	0.7171	0.7020	2
MT_100_	0.6979	0.6554	0.9760	0.9863	0.7599	0.9824	0.9654	0.8605	1
MT_125_	0.2545	0.7735	0.4292	0.7688	0.6529	0.7935	0.7324	0.6292	4
MT_150_	0.8090	0.7482	0.1686	0.7522	0.8477	0.7663	0.6486	0.6772	3
MT_200_	0.3299	0.3046	0.1825	0.5491	0.4355	0.5543	0.2713	0.3753	7

## 4 Discussion

### 4.1 Effects of different concentrations of melatonin on seed germination and seedling growth indexes of millet varieties

Seed germination represents a pivotal phase in the plant life cycle, with rapid germination in dry conditions being vital for plant adaptation to drought and subsequent growth (Qian et al., 2020). External application of melatonin has been proven to effectively boost the vitality of alfalfa (*Medicago sativa*) (Zhao, 2022) and cotton (*Gossypium hirsutum*) (Xiao et al., 2019). For example, Li et al. (2022) demonstrated that suitable concentrations of melatonin significantly enhanced germination characteristics and drought tolerance in millet seeds. In a similar vein, other studies have observed that a 100 μmol/L melatonin solution can increase the antioxidant capacity of aging Bromus inermis seeds, subsequently improving their germination rate, germination index and seedling vitality index (Sun et al., 2019).

The findings of this study suggest that melatonin, at varying concentrations, exhibits a similar impact on the germination of millet seeds. Lower concentrations of melatonin were found to enhance seed germination, while higher concentrations inhibited it. Under the MT100 treatment, Yugu 18 and Jigu 38 seeds achieved their optimal levels in terms of germination rate, germination potential, germination index, and vigor index. For Changnong 35 seeds, the most significant improvement was observed under the MT100 treatment, with performance declining as the solution concentration increased. It is noteworthy that the germination parameters of Yugu 18, Jigu 38, and Changnong 35 all reached their nadir under the MT200 treatment. These observations align broadly with the findings of previous studies, reinforcing the dualistic role of melatonin in modulating seed germination based on its concentration.

Drought exerts profound effects on plant growth and development, and the application of exogenous melatonin has been shown to alleviate growth inhibition induced by drought stress, thereby bolstering plants’ resilience (Wang et al., 2012). Ye et al. (2015) found that applying exogenous melatonin to the roots significantly increased the root-to-shoot ratio of wheat (*Triticum aestivum*) subjected to drought stress. Ma et al. (2021) examined the root system and drought resistance of maize seedlings using two distinct melatonin application techniques: soil drenching and seed soaking. Their research revealed that melatonin markedly extended the seeding and root lengths of Yugu 18, Jigu 38, and Changnong 35 varieties. These observations corroborate the results of our study, indicating that varying concentrations of melatonin can mitigate stress inhibitory effects to different extents (Liu et al., 2015). The variations in seedling growth indices among different millet varieties might be due to the fact that melatonin and auxin share common precursor substances and exhibit overlapping physiological functions. Melatonin can stimulate auxin synthesis, thereby enhancing physiological and metabolic activities in maize seedlings. This process mitigates the toxicity induced by drought stress, promoting normal growth and development of millet seedlings and their roots. Therefore, administering a specific concentration of melatonin under drought stress conditions is crucial for millet root growth.

### 4.2 Effects of different concentrations of melatonin on photosynthesis of millet varieties

Photosynthesis is a critical process necessary for plant viability, yet it can be inhibited by drought stress-induced reactive oxygen species that degrade leaf chlorophyll, thereby diminishing photosynthetic efficiency and function (Huang et al., 2018 and Zhang et al., 2015). However, under drought conditions, the application of melatonin has been demonstrated to alleviate these effects. Melatonin can enhance stomatal conductance and intercellular CO_2_ concentration, thereby decreasing CO_2_ transfer resistance and augmenting CO_2_ absorption rates. Moreover, it optimizes light energy utilization in leaves and safeguards photosynthetic organs from light-induced damage (Yang, 2019).

This experiment revealed that drought stress significantly reduced the photosynthetic and transpiration rates of Yugu 18, Jigu 38, and Changnong 35 millet varieties. Concurrently, there was an increase in stomatal conductance, intercellular CO_2_ concentration, and chlorophyll content. The application of 75 μmol·L^-1^ melatonin notably decreased the photosynthetic rate, intercellular CO_2_ concentration, and chlorophyll content in Jigu 38. However, for Yugu 18 and Changnong 35, these parameters reached their peak after treatment with 100 μmol·L^-1^ melatonin. Furthermore, the stomatal conductance and transpiration rate of Jigu 38 achieved their highest levels under this treatment, demonstrating statistical significance.

Further analysis indicated that a rise in melatonin concentration resulted in a significant reduction in the photosynthetic rate, intercellular CO_2_ concentration, transpiration rate and chlorophyll content in Yugu 18, Jigu 37, and Changnong 35 millet varieties. Conversely, there was a slight increase in stomatal conductance. These findings suggest that an optimal melatonin concentration can substantially enhance the net photosynthetic rate, stomatal conductance, and transpiration rate under drought conditions (Qi et al., 2021). This allows plants to reopen their stomata, thereby improving stomatal function, a phenomenon previously observed in various plant species such as apple (Li et al., 2015), soybean (Zhang et al., 2021) and cucumber (Li et al., 2017).

### 4.3 Effects of different concentrations of melatonin on physiological characteristics of millet varieties

Drought stress can alter the content and activity of endogenous hormones within plants, thereby regulating their physiological and biochemical processes, as well as the transport and distribution of assimilation products (Farooq et al., 2009). This stress disrupts the balance of reactive oxygen species (ROS) in plants, leading to an increase in ROS concentration. Subsequently, this results in cell membrane damage and malondialdehyde production, causing irreversible cellular harm. Additionally, the synthesis of soluble protein within the plants is inhibited (Liu, 2021). Melatonin plays a crucial role in mitigating these effects by scavenging reactive oxygen species and maintaining the metabolic balance of ROS. It reduces the damage caused by ROS to the cell membrane system while enhancing the activity of antioxidant enzymes to eliminate excessive ROS (He et al., 2019). This study found that different concentrations of melatonin exhibited stress-resistance effects on millet varieties, with 75 and 100 μmol·L^-1^ melatonin proving to be the most effective. After treatment with 75 μmol·L^-1^ melatonin, there was a significant increase in the MDA content in Changnong 35 seedlings, SOD activity in Yugu 18 seedlings, and CAT activity in Jigu 38 seedlings. A 100 μmol·L^-1^ melatonin treatment led to a notable rise in CAT activity in Jigu 38 seedlings. Furthermore, the activities of soluble protein, CAT, and POD in Yugu 18 and Changnong 35 seedlings were significantly enhanced, along with an increase in the activities of SOD and MDA. However, the levels of ASA and GSH decreased significantly. The reduction in ASA and GSH levels during drought stress primarily stems from their increased consumption to mitigate the excessive ROS produced by such conditions. This consumption can exceed their replenishment capabilities. Additionally, there is an inhibition of their biosynthesis pathways as plants reallocate resources to other stress-response mechanisms, leading to a decreased supply of precursors and energy for synthesis. Principal component analysis revealed that the five most influential factors were POD activity, Ci, GSH content, soluble protein content, and SOD activity, listed in descending order. The experiment also demonstrated that exogenous melatonin spraying at a certain concentration aided in scavenging active oxygen species and peroxide in millet, thereby maintaining cell membrane stability.Notably, however, an increased concentration of melatonin did not demonstrate a significant effect, which aligns with the findings of Yang et al (Yang et al., 2017). The utilization of melatonin can bolster the antioxidant and water-holding capacities of millet, thereby enhancing photosynthesis and substantially augmenting the drought and stress resistance of crops (Fleta-Soriano et al., 2017).

## 5 Conclusion

The drought stress environment significantly affects the seedling development of assorted millet varieties. However, the application of melatonin can moderate this detrimental impact on millet germination to a certain degree, albeit with varying results depending on the concentration used and the variety of the millet. A concentration that is too high can hinder seed germination. The Jigu 38 variety, when treated with 75 μmol·L^-1^ melatonin, demonstrated superior drought resistance. Similarly, Yugu 18 and Changnong 35 varieties, when treated with 100 μmol·L^-1^ melatonin, effectively modulated the antioxidant enzyme activity and osmotic regulation ability of millet seedlings, improved leaf photosynthesis, and mitigated the effects of drought stress on millet seedling growth and development. Hence, the strategic application of melatonin substantially enhances the drought resistance of millet varieties. This provides a theoretical foundation for further research into substances that boost the drought resistance of millet and their respective mechanisms of action.

## Data Availability

The original contributions presented in the study are included in the article/**Supplementary Material**. Further inquiries can be directed to the corresponding authors.
