# Supplementary material for: Effects of melatonin treatment on germination, growth and physiological characteristics under drought stress in foxtail millet
Source: Front Plant Sci. 2025 Jun 6;16:1601253. doi: 10.3389/fpls.2025.1601253 (PMC12179618; doi:10.3389/fpls.2025.1601253)
Supplement: Supplementary file 1 [file DataSheet1.docx]

Supplementary Material

**Figures, tables, and images**

TABLE1 Effects of different concentrations of melatonin on seed germination in millet varieties

| variety | content | Determination index | | | | Allelopathy index | | | | |
| --- | --- | --- | --- | --- | --- | --- | --- | --- | --- | --- |
|  |  | GR(%) | GP(%) | GI(%) | VI(%) | GR | GP | GI | VI | SE |
| Yugu 18 | CK | 23.33±2.67c | 17.67±1.45c | 15.99±2.35b | 49.28±4.93c |  |  |  |  |  |
|  | MT_5_ | 22.67±0.66b | 19.33±0.88c | 16.57±1.29b | 49.67±12.13c | -0.03 | 0.09 | 0.04 | 0.01 | 0.03 |
|  | MT_25_ | 24.33±1.20b | 22.67±0.88b | 18.14±0.50b | 48.41±2.03c | 0.04 | 0.15 | 0.12 | -0.02 | 0.07 |
|  | MT_50_ | 25.67±0.33b | 23.67±0.67b | 18.99±2.23b | 55.25±1.87b | 0.09 | 0.04 | 0.16 | 0.11 | 0.10 |
|  | MT_75_ | 26.33±0.33c | 24.67±0.33b | 22.42±3.53b | 65.48±2.02c | 0.11 | 0.01 | 0.29 | 0.25 | 0.17 |
|  | MT_100_ | 28.33±0.67c | 26.67±0.88c | 23.38±1.16b | 72.71±12.74b | 0.18 | 0.10 | 0.32 | 0.32 | 0.23 |
|  | MT_125_ | 25.67±0.67c | 23.33±0.88c | 21.00±3.51c | 69.61±4.90c | 0.09 | 0.14 | 0.24 | 0.29 | 0.19 |
|  | MT_150_ | 24.33±2.03c | 20.67±1.52c | 14.14±3.45c | 43.11±11.18b | 0.04 | 0.13 | -0.13 | -0.14 | -0.03 |
|  | MT_200_ | 22.33±1.45c | 18.67±1.20c | 10.85±1.50c | 30.43±5.18b | -0.04 | 0.11 | -0.47 | -0.62 | -0.26 |
| Jigu 38 | CK | 31.66±1.20b | 27.67±2.40b | 24.28±2.86b | 75.19±0.96b |  |  |  |  |  |
|  | MT_5_ | 41.67±2.03a | 29.33±4.26a | 25.95±1.58b | 77.06±1.38b | 0.24 | 0.06 | 0.06 | 0.02 | 0.10 |
|  | MT_25_ | 42.33±2.33a | 33.67±1.67a | 31.04±0.33b | 81.21±10.51b | 0.25 | 0.18 | 0.22 | 0.07 | 0.18 |
|  | MT_50_ | 45.67±4.41a | 40.67±2.40a | 33.52±1.53a | 87.89±4.81ab | 0.31 | 0.32 | 0.28 | 0.14 | 0.26 |
|  | MT_75_ | 46.33±3.28b | 44.33±2.33b | 35.62±0.96a | 106.23±0.61b | 0.32 | 0.38 | 0.32 | 0.29 | 0.33 |
|  | MT_100_ | 54.33±3.38b | 47.33±3.18a | 37.76±0.81b | 128.83±4.82a | 0.42 | 0.42 | 0.36 | 0.42 | 0.41 |
|  | MT_125_ | 41.67±3.17b | 41.67±0.88b | 33.61±1.77b | 116.94±4.20b | 0.24 | 0.34 | 0.28 | 0.36 | 0.31 |
|  | MT_150_ | 40.33±0.88b | 40.33±0.88b | 31.76±1.12b | 118.90±5.34a | 0.21 | 0.31 | 0.24 | 0.37 | 0.28 |
|  | MT_200_ | 38.33±3.48b | 38.33±1.20b | 27.14±1.44b | 57.62±4.83b | 0.17 | 0.28 | 0.11 | -0.30 | 0.07 |
| Changnong 35 | CK | 47.67±1.21d | 40.33±0.67a | 33.14±1.78a | 103.65±8.66a |  |  |  |  |  |
|  | MT_5_ | 39.33±1.76a | 35.67±1.20a | 33.95±0.70a | 104.63±1.91a | -0.21 | -0.13 | 0.02 | 0.01 | -0.08 |
|  | MT_25_ | 40.67±2.33a | 36.67±2.73a | 36.14±1.36a | 121.82±7.62a | -0.17 | -0.10 | 0.08 | 0.15 | -0.01 |
|  | MT_50_ | 42.33±1.76a | 39.67±0.88a | 38.05±1.27a | 125.52±3.34a | -0.13 | -0.02 | 0.13 | 0.17 | 0.04 |
|  | MT_75_ | 60.33±3.18a | 49.33±0.88a | 42.62±2.19a | 131.69±3.72a | 0.21 | 0.18 | 0.22 | 0.21 | 0.21 |
|  | MT_100_ | 62.67±1.20a | 53.33±1.85a | 45.95±1.68a | 139.70±6.23a | 0.24 | 0.24 | 0.28 | 0.26 | 0.26 |
|  | MT_125_ | 80.67±2.19a | 69.33±1.45a | 50.86±2.41a | 163.93±9.76a | 0.41 | 0.42 | 0.35 | 0.37 | 0.39 |
|  | MT_150_ | 63.33±1.20a | 50.33±0.66a | 48.38±7.37b | 154.55±14.15a | 0.25 | 0.20 | 0.32 | 0.33 | 0.28 |
|  | MT_200_ | 55.67±0.88a | 45.67±1.20a | 44.61±2.97a | 133.15±11.34a | -0.14 | -0.12 | 0.26 | 0.22 | 0.06 |

Note：GR: Germination rate; GP: Germination potential; GI: Germination index; VI: Vitality index; Vitality Index; Different lowercase letters in the same column indicate significant differences (*P*<0.05). Different lowercase letters in the same column indicate significant differences between different varieties and concentrations (*P*<0.05).

TABLE 2 Effects of different concentrations of melatonin on bud length, seedling length and root length of millet under drought stress

| variety | content | Germination index | | Pot index | |
| --- | --- | --- | --- | --- | --- |
|  |  | BG | RL | SL | RL |
| Yugu 18 | CK | 30.24±2.06a | 49.20±2.72b | 138.61±6.45a | 124.21±2.22a |
|  | MT_5_ | 29.40±9.33a | 49.09±4.21b | 117.62±5.44a | 98.61±3.53a |
|  | MT_25_ | 26.68±1.38a | 61.84±5.38a | 107.35±4.75a | 148.14±3.66a |
|  | MT_50_ | 19.67±5.12a | 74.54±1.36a | 127.97±5.89a | 157.56±6.22a |
|  | MT_75_ | 24.52±1.57a | 79.88±4.06a | 139.37±7.22a | 171.81±5.34a |
|  | MT_100_ | 34.70±8.58a | 84.83±2.23a | 165.05±3.70a | 191.14±3.42a |
|  | MT_125_ | 31.82±7.44a | 59.58±4.82b | 148.38±5.87ab | 175.44±9.01a |
|  | MT_150_ | 30.47±4.29a | 74.91±3.95a | 139.38±7.38a | 185.30±0.62a |
|  | MT_200_ | 27.74±1.86a | 49.65±2.78b | 144.25±7.56a | 172.92±6.69a |
| Jigu 38 | CK | 21.42±2.88b | 68.83±3.69a | 124.47±2.67b | 152.07±5.01a |
|  | MT_5_ | 18.70±2.10a | 74.72±4.82a | 92.67±10.30b | 141.26±4.40b |
|  | MT_25_ | 23.93±6.53a | 62.42±6.61a | 117.31±8.61a | 137.50±3.11a |
|  | MT_50_ | 25.93±8.84a | 65.45±5.11b | 134.53±9.30b | 153.37±3.45a |
|  | MT_75_ | 32.63±4.74b | 85.15±3.36a | 135.40±3.82a | 165.57±3.03a |
|  | MT_100_ | 39.42±1.67a | 87.68±3.92a | 145.58±5.13a | 185.60±0.04a |
|  | MT_125_ | 35.05±4.85a | 86.40±5.01a | 127.15±4.39c | 174.03±6.77a |
|  | MT_150_ | 37.47±2.62a | 65.80±2.31a | 118.09±4.93b | 161.48±7.66a |
|  | MT_200_ | 34.43±2.19a | 84.44±4.43b | 121.05±4.25b | 176.28±4.80a |
| Changnong 35 | CK | 31.17±1.71a | 47.49±5.14b | 93.54±5.83c | 156.25±0.07a |
|  | MT_5_ | 29.71±2.87a | 40.49±2.72c | 86.26±8.79b | 140.73±2.76b |
|  | MT_25_ | 25.47±1.15a | 30.48±5.57a | 120.33±8.54a | 129.09±6.20a |
|  | MT_50_ | 22.60±1.00a | 36.12±4.56c | 132.49±5.46a | 143.16±1.26a |
|  | MT_75_ | 20.16±0.45a | 34.08±7.60b | 138.65±4.97a | 152.96±5.54a |
|  | MT_100_ | 30.26±4.45a | 42.67±3.67b | 143.29±8.48b | 163.81±1.08a |
|  | MT_125_ | 33.06±1.68a | 48.92±4.49c | 154.71±10.69a | 205.98±1.27a |
|  | MT_150_ | 32.04±0.71a | 46.37±6.49b | 97.62±3.72c | 183.29±4.08a |
|  | MT_200_ | 29.79±1.61a | 42.96±5.49a | 119.03±5.98b | 170.31±3.82a |

Note: BG:Bud growth; SL:Seedling length; RL:Root length; Different lowercase letters in the same column indicate significant differences (P< 0.05). Different lowercase letters in the same column indicate significant differences between different varieties and concentrations (P< 0.05).


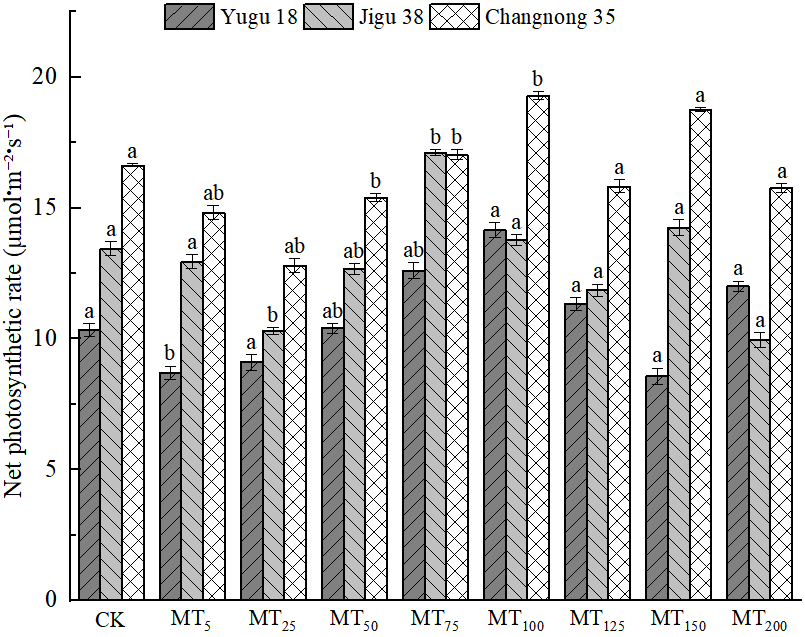

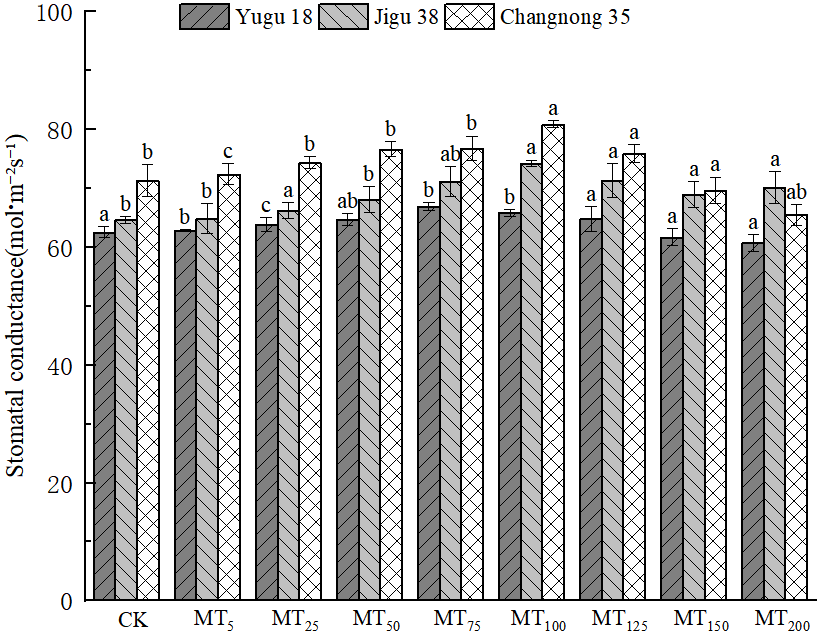


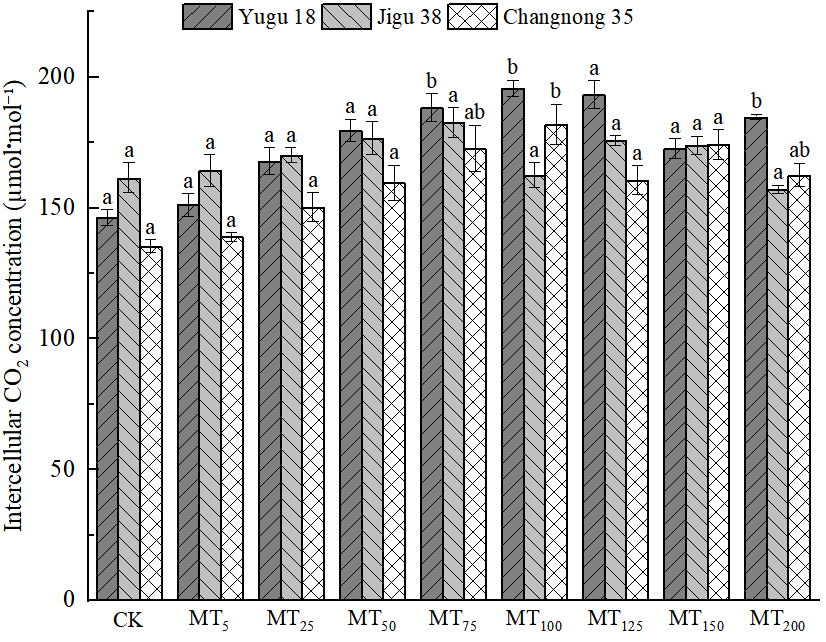

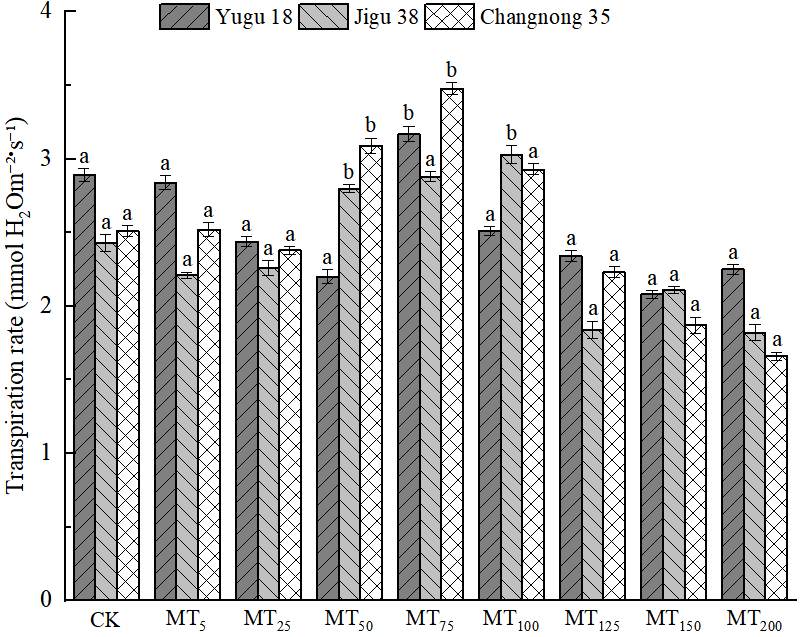


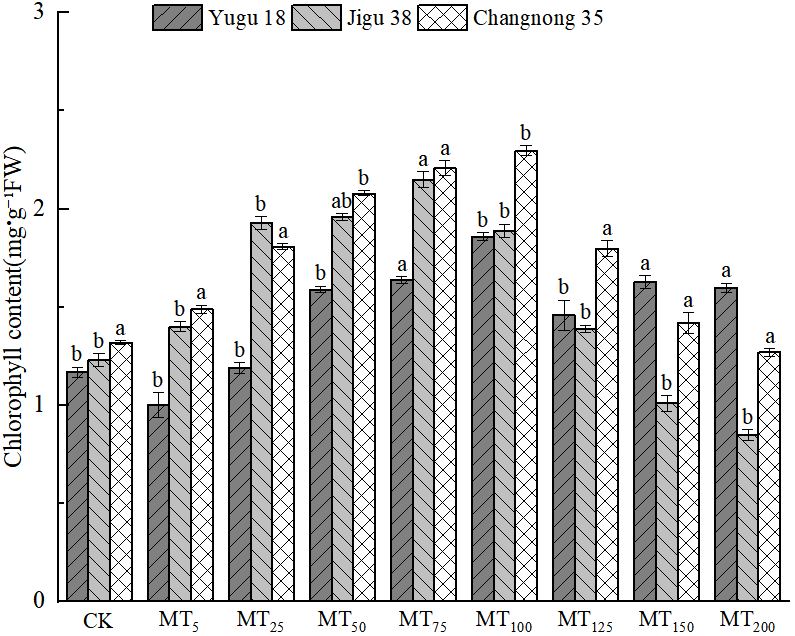


FIGURE 1 Effects of different concentrations of melatonin on photosynthetic parameters of millet seedlings under drought stress


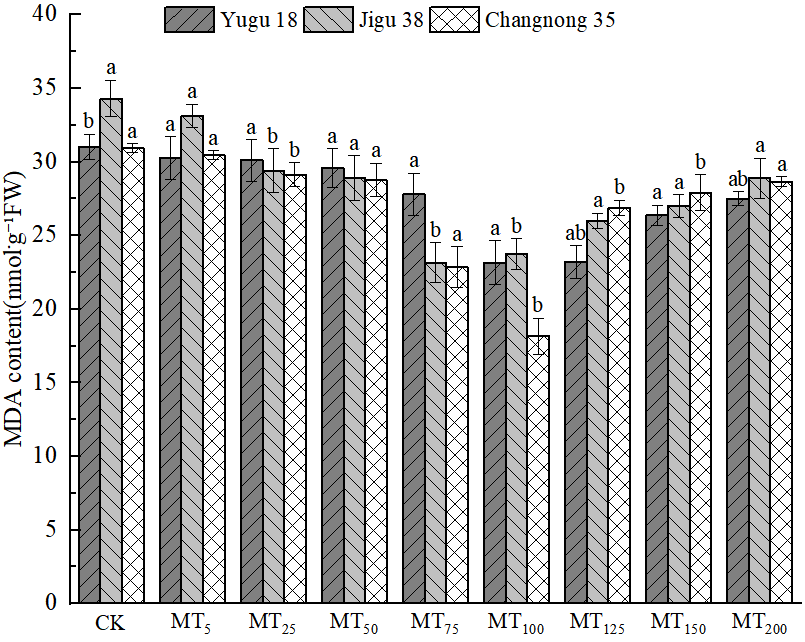


FIGURE 2 Effects of different concentrations of melatonin on MDA content of millet seedlings under drought stress


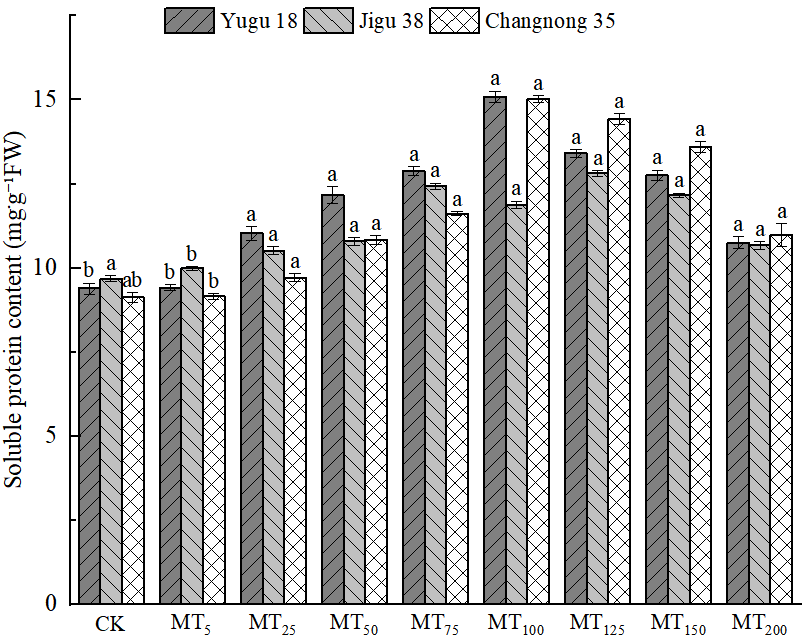


Figure 3 Effects of different concentrations of melatonin on soluble protein content of millet seedlings under drought stress


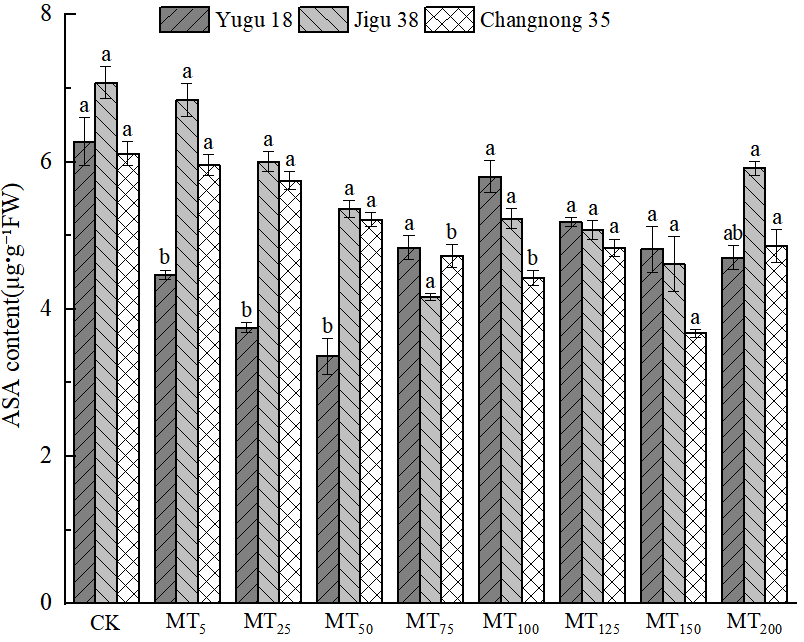

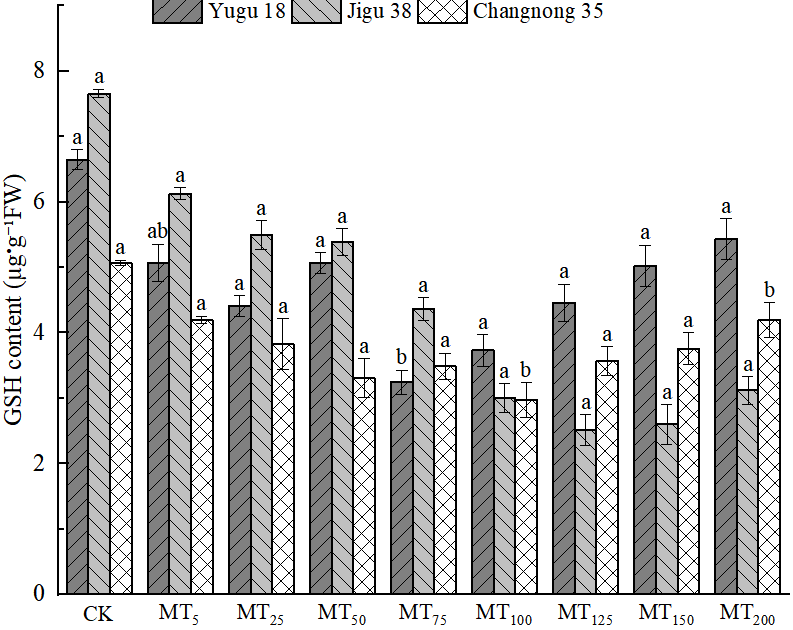


Figure 4 Effects of different concentrations of melatonin on ASA and GSH contents of millet seedlings under drought stress


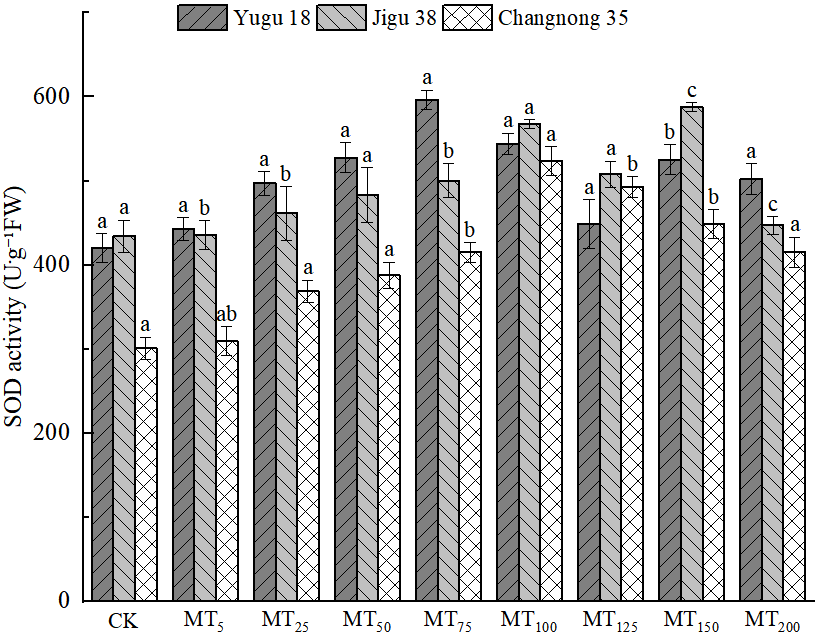

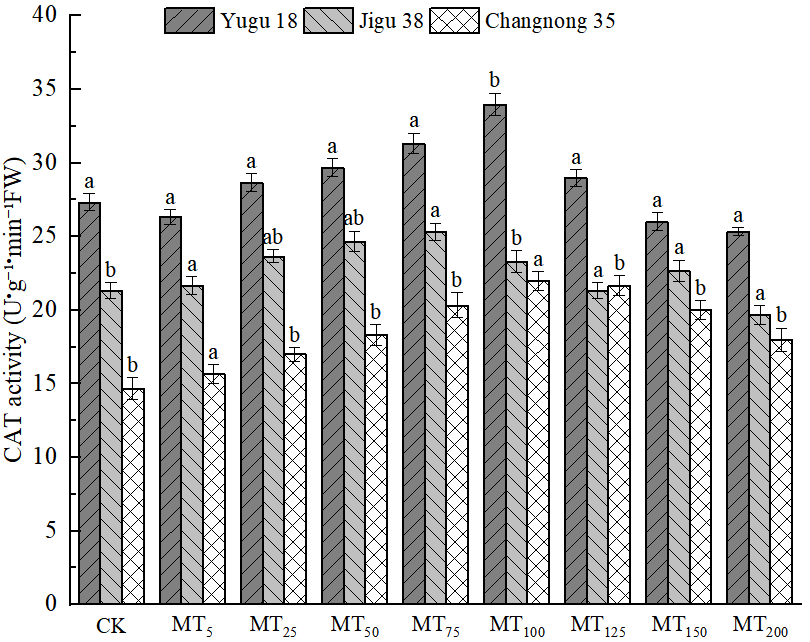


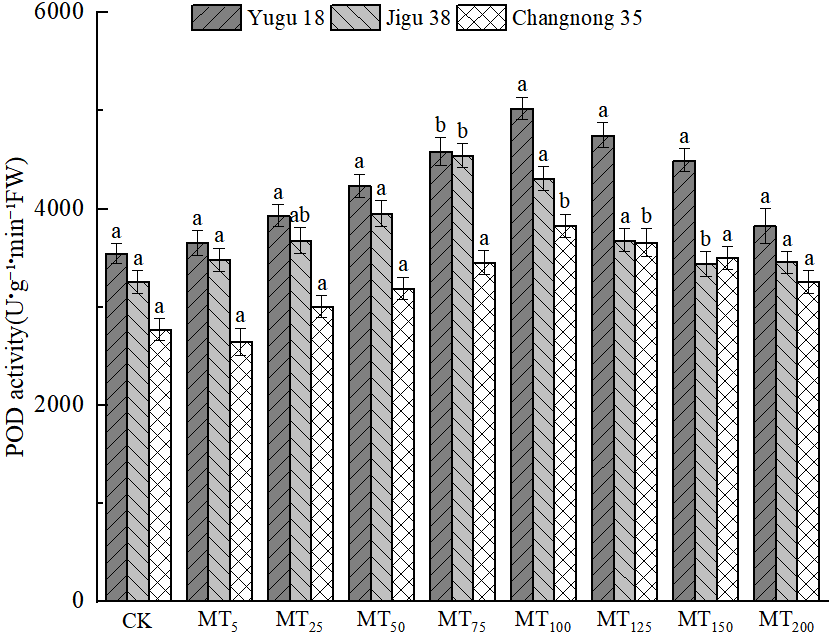


FIGURE 5 Effects of different concentrations of melatonin on antioxidant oxidase activities of millet seedlings under drought stress


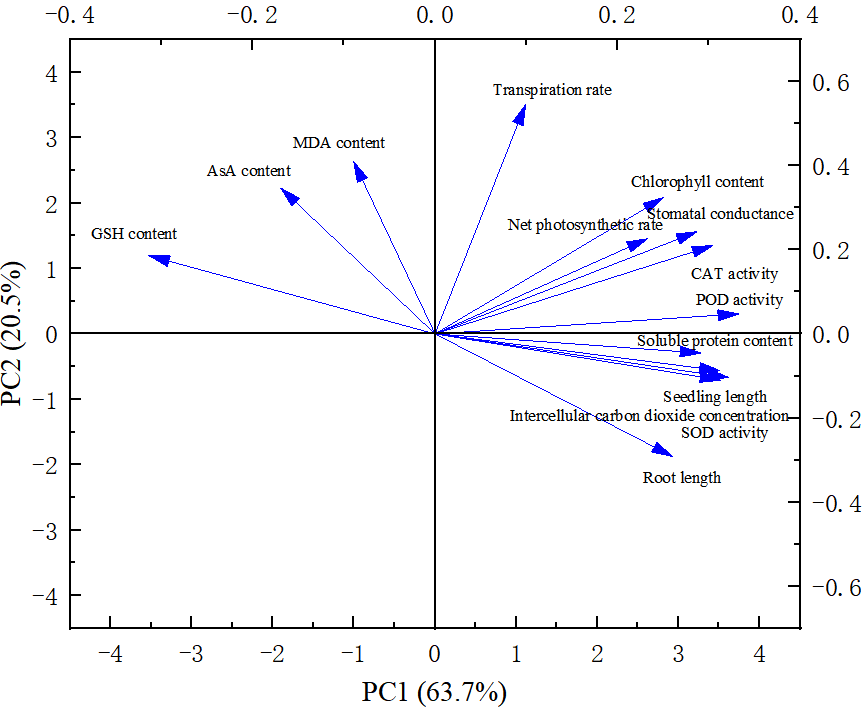


FIGURE 6 Principal component analysis of each index under drought stress

TABLE 3 Component load matrix, eigenvector and weight analysis of millet indexes under drought stress

| Parameter | principal component 1 | | principal component 2 | | principal component 3 | | Weight(%) | Influence order |
| --- | --- | --- | --- | --- | --- | --- | --- | --- |
|  | Eigenvector | Loading matri（*x*） | Eigenvector | Loading matri（*x*） | Eigenvector | Loading matri（*x*） |  |  |
| Seedling length | 0.291 | 0.87 | -0.045 | -0.077 | -0.121 | -0.122 | 11.99 | 7 |
| root length | 0.260 | 0.777 | -0.291 | -0.493 | 0.218 | 0.22 | 10.71 | 9 |
| photosynthetic rate | 0.233 | 0.696 | 0.227 | 0.384 | 0.517 | 0.523 | 9.59 | 11 |
| stomatal conductance | 0.287 | 0.858 | 0.243 | 0.412 | -0.270 | -0.273 | 11.82 | 8 |
| intercellular CO_2_ concentration | 0.322 | 0.96 | -0.103 | -0.175 | 0.029 | 0.029 | 13.23 | 2 |
| transpiration rate | 0.099 | 0.297 | 0.545 | 0.924 | -0.035 | -0.035 | 4.07 | 13 |
| chlorophyll content | 0.251 | 0.748 | 0.324 | 0.549 | -0.317 | -0.32 | 10.30 | 10 |
| MAD content | -0.089 | -0.266 | 0.410 | 0.695 | 0.623 | 0.63 | 3.70 | 14 |
| Soluble protein content | 0.313 | 0.935 | -0.110 | -0.186 | 0.063 | 0.064 | 12.89 | 4 |
| ASAcontent | -0.169 | -0.505 | 0.347 | 0.588 | -0.194 | -0.196 | 7.01 | 12 |
| GSH content | -0.314 | -0.937 | 0.186 | 0.315 | 0.002 | 0.002 | 12.97 | 3 |
| SOD activity | 0.313 | 0.933 | -0.087 | -0.148 | 0.204 | 0.206 | 12.86 | 5 |
| CAT activity | 0.304 | 0.909 | 0.210 | 0.355 | -0.154 | -0.156 | 12.53 | 6 |
| PODactivity | 0.333 | 0.993 | 0.047 | 0.08 | -0.013 | -0.013 | 13.69 | 1 |


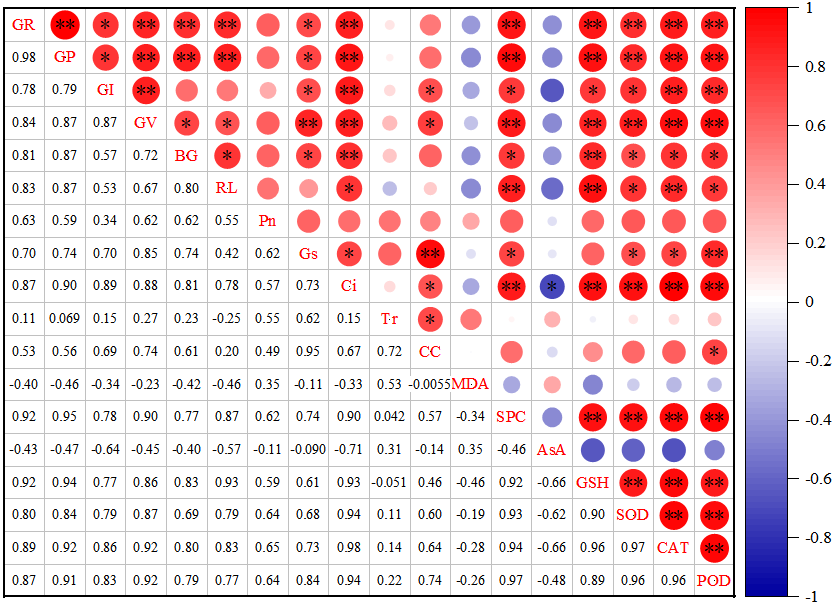


FIGURE 7 Correlation analysis of each index under drought stress

TABLE 4 Average membership function values of millet physiological indexes under drought stress

| Treatment | MAD content | Soluble protein content | ASAcontent | GSH content | SOD activity | CAT activity | POD  activity | Average value | Rank |
| --- | --- | --- | --- | --- | --- | --- | --- | --- | --- |
| CK | 0.9349 | 0.4245 | 0.6521 | 0.3113 | 0.0858 | 0.2241 | 0.3134 | 0.4209 | 6 |
| MT_5_ | 0.9584 | 0.0277 | 0.5132 | 0.2741 | 0.0681 | 0.1329 | 0.0218 | 0.2852 | 9 |
| MT_25_ | 0.2840 | 0.2216 | 0.4819 | 0.2819 | 0.3579 | 0.3315 | 0.3273 | 0.3266 | 8 |
| MT_50_ | 0.8231 | 0.4067 | 0.2784 | 0.3844 | 0.5072 | 0.5761 | 0.5318 | 0.5011 | 5 |
| MT_75_ | 0.8099 | 0.6330 | 0.2506 | 0.8576 | 0.7437 | 0.9022 | 0.7171 | 0.7020 | 2 |
| MT_100_ | 0.6979 | 0.6554 | 0.9760 | 0.9863 | 0.7599 | 0.9824 | 0.9654 | 0.8605 | 1 |
| MT_125_ | 0.2545 | 0.7735 | 0.4292 | 0.7688 | 0.6529 | 0.7935 | 0.7324 | 0.6292 | 4 |
| MT_150_ | 0.8090 | 0.7482 | 0.1686 | 0.7522 | 0.8477 | 0.7663 | 0.6486 | 0.6772 | 3 |
| MT_200_ | 0.3299 | 0.3046 | 0.1825 | 0.5491 | 0.4355 | 0.5543 | 0.2713 | 0.3753 | 7 |
